# ACC1 is a dual metabolic-epigenetic regulator of Treg stability and immune tolerance

**DOI:** 10.1016/j.molmet.2025.102111

**Published:** 2025-02-08

**Authors:** Philipp Stüve, Gloria J. Godoy, Fernando N. Ferreyra, Florencia Hellriegel, Fatima Boukhallouk, Yu-San Kao, Tushar H. More, Anne-Marie Matthies, Tatiana Akimova, Wolf-Rainer Abraham, Volkhard Kaever, Ingo Schmitz, Karsten Hiller, Matthias Lochner, Benoît L. Salomon, Ulf H. Beier, Michael Rehli, Tim Sparwasser, Luciana Berod

**Affiliations:** 1 Institute of Infection Immunology, TWINCORE, Centre for Experimental and Clinical Infection Research, Germany; 2 A Joint Venture Between the Hannover Medical School (MHH) and the Helmholtz Centre for Infection Research (HZI), Hannover 30625, Germany; 3 Leibniz Institute for Immunotherapy, Regensburg, Germany; 4 Institute of Medical Microbiology and Hygiene, University Medical Center of the Johannes Gutenberg-University Mainz, Mainz 55122, Germany; 5 Institute for Molecular Medicine, University Medical Center of the Johannes Gutenberg-University Mainz, Mainz 55131, Germany; 6 Centro de Investigaciones en Bioquímica Clínica e Inmunología (CIBICI), Consejo Nacional de Investigaciones Científicas y Técnicas (CONICET), Córdoba, Argentina; 7 Departamento de Bioquímica Clínica, Facultad de Ciencias Químicas, Universidad Nacional de Córdoba, Córdoba, Argentina; 8 Department of Bioinformatics and Biochemistry, BRICS, Technische Universität Braunschweig, 38106 Braunschweig, Germany; 9 Systems-Oriented Immunology and Inflammation Research Group, Department of Experimental Immunology, HZI, Braunschweig 38124, Germany; 10 Institute for Molecular and Clinical Immunology, Otto-von-Guericke University Magdeburg, Magdeburg 39106, Germany; 11 Institute for Molecular Immunology, Ruhr-University Bochum, Bochum 44801, Germany; 12 Division of Transplant Immunology, Department of Pathology and Laboratory Medicine, Children's Hospital of Philadelphia and University of Pennsylvania, Philadelphia, PA 19104, USA; 13 Department of Chemical Microbiology, HZI, Braunschweig 38124, Germany; 14 Research Core Unit Metabolomics, MHH, Hannover 30625, Germany; 15 Institute of Medical Microbiology and Hospital Epidemiology, MHH, Hannover 30625, Germany; 16 Sorbonne Université, INSERM, CNRS, Centre d’Immunologie et des Maladies Infectieuses (CIMI-Paris), Paris 75013, France; 17 Division of Nephrology and Department of Pediatrics, Children's Hospital of Philadelphia and University of Pennsylvania, Philadelphia, PA 19104, USA; 18 Department of Internal Medicine III, University Hospital Regensburg, Regensburg, Germany; 19 Research Center for Immunotherapy (FZI), University Medical Center Mainz, 55131 Mainz, Germany

**Keywords:** ACC1, Adoptive Treg transfer, Epigenetic regulation, Fatty acid synthesis, Treg stability, Acetylation

## Abstract

**Objective:**

Regulatory T cells (Tregs) are essential in maintaining immune tolerance and controlling inflammation. Treg stability relies on transcriptional and post-translational mechanisms, including histone acetylation at the *Foxp3* locus and FoxP3 protein acetylation. Additionally, Tregs depend on specific metabolic programs for differentiation, yet the underlying molecular mechanisms remain elusive. We aimed to investigate the role of acetyl-CoA carboxylase 1 (ACC1) in the differentiation, stability, and function of regulatory T cells (Tregs).

**Methods:**

We used either T cell-specific ACC1 knockout mice or ACC1 inhibition via a pharmacological agent to examine the effects on Treg differentiation and stability. The impact of ACC1 inhibition on Treg function was assessed *in vivo* through adoptive transfer models of Th1/Th17-driven inflammatory diseases.

**Results:**

Inhibition or genetic deletion of ACC1 led to an increase in acetyl-CoA availability, promoting enhanced histone and protein acetylation, and sustained FoxP3 transcription even under inflammatory conditions. Mice with T cell-specific ACC1 deletion exhibited an enrichment of double positive RORγt^+^FoxP3^+^ cells. Moreover, Tregs treated with an ACC1 inhibitor demonstrated superior long-term stability and an enhanced capacity to suppress Th1/Th17-driven inflammatory diseases in adoptive transfer models.

**Conclusions:**

We identified ACC1 as a metabolic checkpoint in Treg biology. Our data demonstrate that ACC1 inhibition promotes Treg differentiation and long-term stability *in vitro* and *in vivo*. Thus, ACC1 serves as a dual metabolic and epigenetic hub, regulating immune tolerance and inflammation by balancing *de novo* lipid synthesis and protein acetylation.

## Highlights

• ACC1 inhibition, but not ACC2, results in elevated intracellular acetyl-CoA levels during Treg cell differentiation.
• Increased acetyl-CoA levels drive global protein and histone acetylation enhancing histone acetylation at the *Foxp3* locus.
• Fatty acid inhibition promotes the induction and long-term stability of Tregs, even under inflammatory conditions.

## Graphical abstract

**Figure:**
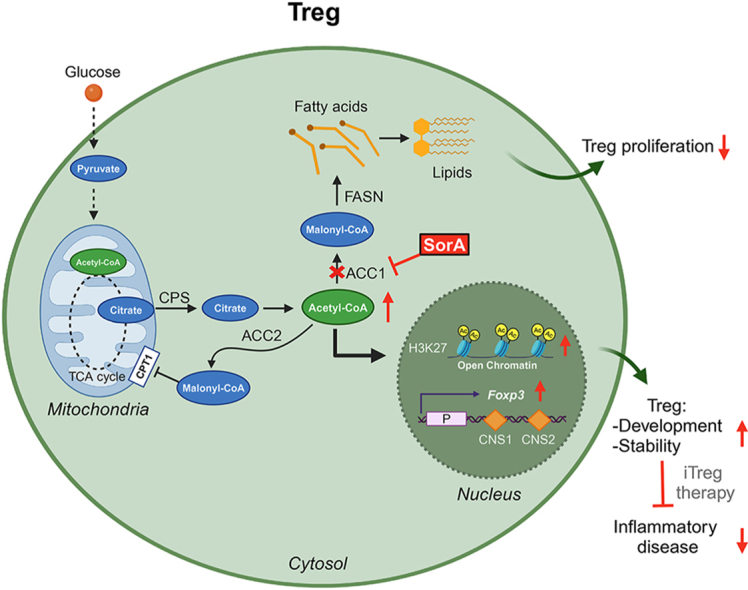

## 1 Introduction

FoxP3^+^ regulatory T cells (Tregs) are characterised by their ability to suppress immune responses and maintain tolerance [[1], [2], [3]]. Most Tregs develop in the thymus (tTregs or nTregs), while some are differentiated from naïve T cells under specific conditions *in vitro* (iTregs) or the periphery *in vivo* (pTregs), in particular in the gut [4,5]. In contrast, IL-17-producing Th17 cells contribute to the host's defence against fungal and extracellular pathogens [6]. Their sustained activation can cause immunopathology associated with autoimmune diseases. Both Treg and Th17 cell lineages display developmental similarities and plasticity between each other. In the absence of inflammation, TGF-β induces the development of FoxP3^+^ Tregs, whereas the additional presence of IL-6 results in the generation of Th17 cells [[7], [8], [9]]. Interestingly, a subset of Tregs, known as RORγt^+^FoxP3^+^ Tregs, express both the Treg master regulator FoxP3 and the Th17-associated transcription factor RORγt. These cells are primarily found in the gut and are thought to play a unique role in maintaining immune tolerance to commensal microbiota while balancing inflammatory responses [10,11].

We and others have previously shown that upon activation, naïve T cells differentiating into T effector lineages shift their metabolism towards the glycolytic-lipogenic pathway [[12], [13], [14], [15]]. Cytosolic Acetyl-CoA-carboxylase 1 (ACC1) catalyses the ATP-dependent carboxylation of acetyl-CoA to malonyl-CoA, the rate-limiting step in the synthesis of fatty acids (FAS). Pharmacological inhibition of ACC1 with the myxobacterial metabolite Soraphen A (SorA) or genetic deletion of ACC1 in T cells restrains Th17 but promotes Treg development [12]. Tregs are less dependent on the glycolytic-lipogenic route. Instead, it has been suggested that FoxP3 directly drives mitochondrial oxidative phosphorylation (OXPHOS) to meet the energetic demands of development and function [[16], [17], [18], [19], [20]]. OXPHOS can be efficiently fuelled by fatty acid oxidation (FAO), a process controlled by ACC2, another isoform of ACC that catalyses the same enzymatic reaction as ACC1, but is located in the outer mitochondrial membrane [21]. FAO has initially been proposed as crucial for Tregs, yet we recently showed that *in vivo* this pathway is dispensable for Treg development and function [22]. Still, whether ACC2 contributes to FAS in the absence of ACC1 remains unclear.

Acetyl-CoA constitutes not only the primary building block for *de novo* FAS but also serves as a substrate for lysine acetylation of histones and extra-nuclear proteins. Previous studies in non-immune cells, such as cancer cells [23], hepatocytes [24], and yeast [25], reported that blocking or deleting ACC1/2 leads to the hyperacetylation of histones and extra-nuclear proteins. While histone acetylation renders chromatin more open, enabling gene transcription [26], protein acetylation promotes protein stability. Acetylation constitutes a reversible process controlled by histone acetyltransferases (HATs; also known as lysine acetyltransferases (KATs)) and histone deacetylases (HDACs; also known as lysine deacetyltransferases (KDACs)). Along with demethylation of the *Foxp3* promoter [[27], [28], [29]] and the Treg cell-specific demethylated region (TSDR) [30], histone acetylation in the *Foxp3* gene locus [[31], [32], [33], [34]] represents a requirement for the development as well as stability of FoxP3^+^ Tregs. In addition to this epigenetic regulation, Treg stability is regulated by post-translational acetylation of the FoxP3 protein [[35], [36], [37]]. In this study, we investigated the mechanisms by which disrupting acetyl-CoA carboxylation affects Treg induction and stability.

Our findings underscore the pivotal role of metabolic regulation in immune cell function, revealing that inhibition of ACC1 during T cell differentiation results in elevated intracellular acetyl-CoA levels, which drive global protein and histone acetylation. These histone modifications are crucial for regulating the transcription of FoxP3. Notably, we observed significantly enhanced *Foxp3* mRNA transcription and increased FoxP3 protein expression, even under inflammatory conditions. These results emphasize a critical connection between metabolic shifts and the induction and stabilization of FoxP3^+^ Tregs, promoting their long-term stability and functional integrity, which has profound implications for immune regulation and therapy.

## 2 Results

### 2.1 Bimodal effect of FAS inhibition on Th17 and Treg development

**Figure 1:**
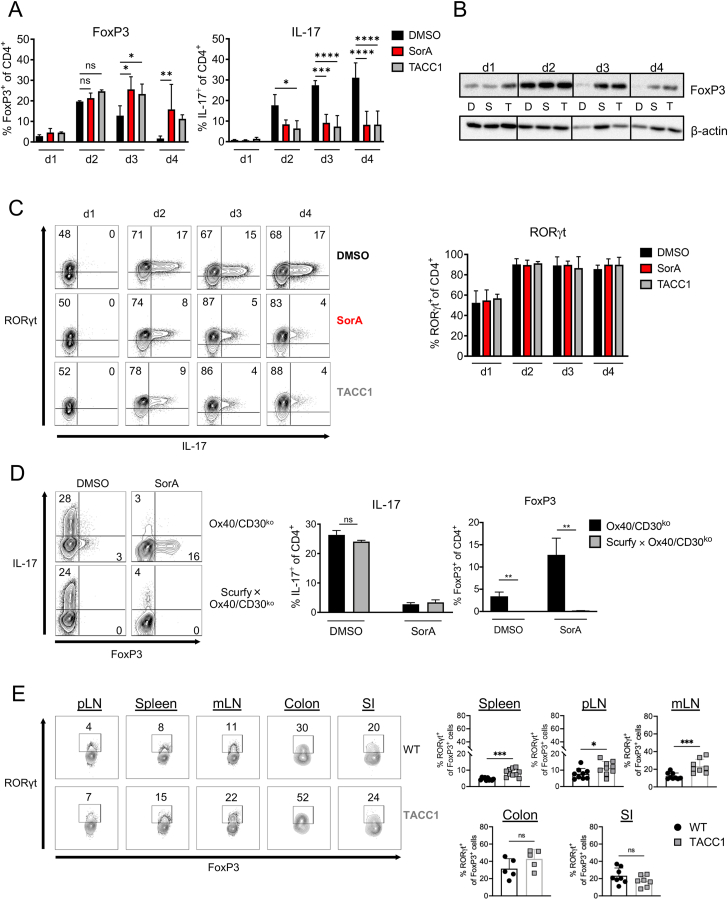
**Bimodal effect of FAS inhibition on Th17 and Treg development.** (A–C) Naïve CD4^+^ T cells from WT and TACC1 mice were cultured under Th17-polarizing conditions in the presence of DMSO or SorA and analysed at different time points. (A) Graphs show frequencies of IL-17^+^ and FoxP3^+^ cells among live CD4^+^ T cells determined by flow cytometry. (B) FoxP3 protein was determined in whole-cell lysates by western blot. β-actin served as a loading control. D: WT cells DMSO-treated, S: WT cells SorA-treated, T: TACC1 cells untreated. (C) Representative flow cytometry depicts the frequency of RORγt^+^ and IL-17^+^ cells among live CD4^+^ T cells. Bar graphs represent the frequency of RORγt^+^ cells among live CD4^+^ T cells. (D) Naïve CD4^+^ T cells from Ox40/CD30^ko^ and Scurfy × Ox40/CD30^ko^ mice were cultured under iTreg- or Th17-polarizing conditions in the presence of DMSO or SorA. Representative flow cytometry shows the frequency of IL-17^+^ and FoxP3^+^ cells in Th17 cultures among live CD4^+^ T cells determined on day 4. Bar graphs represent the frequency of IL-17^+^ and FoxP3^+^ T cells in Th17 cultures. (E) Immune cells were isolated from the spleen, a pool of axillary and inguinal lymph nodes (pLN), mesenteric lymph nodes (mLN), and the colonic and small intestinal (SI) lamina propria, and subjected to further analysis by flow cytometry. Representative contour plots show the frequency of RORγt^+^ FoxP3^+^ among live Treg cells in the indicated organs. Results are representative of two (B) or three independent experiments (D) or shown as pooled data from one (E: colon), two (E: Spleen, pLN, mLN, SI) with n = 7–9 (E: spleen, pLN, mLN, SI) or n = 5 (E: colon) mice per group, or three (C) independent experiments. Each symbol represents an individual mouse. Error bars show s.d. of triplicates (D) or pooled data (A,C,E). ∗*P* < 0.05, ∗∗*P* < 0.01, ∗∗∗*P* < 0.001, ∗∗∗∗P < 0.0001. n.s., non-significant. One-way ANOVA with Bonferroni correction (D) or Two-way ANOVA with Bonferroni correction (A, C) or Two-tailed Mann–Whitney t-test (E).

We have previously demonstrated that targeting ACC1 by T cell-specific deletion (TACC1 mice) or pharmacological inhibition by SorA shifts Th17 toward Treg development [12]. To gain a deeper insight into the mechanisms underlying the induction of FoxP3^+^ cells by ACC1 inhibition, naïve T cells were cultured under Th17-polarising conditions and the expression of IL-17 and FoxP3 proteins was monitored over time using flow cytometry and western blot analysis. After 2 days of culture, the frequency of FoxP3^+^ cells and levels of FoxP3 protein were comparable in the presence or absence of ACC1 activity, both in the SorA-treated and TACC1 group (Figure 1A,B). On days 3 and 4 of culture, lack of ACC1 activity maintained the frequency of FoxP3^+^ cells, which dropped in vehicle-treated WT Th17 cells (Figure 1A,B). In contrast, the frequency of IL-17^+^-producing cells significantly decreased from day 2 onwards in both the SorA-treated and TACC1 groups, compared to the DMSO control (Figure 1A).

Th17 development critically depends on the expression and function of the transcription factor retinoic acid-related orphan receptor-γt (RORγt) [38]. We have previously demonstrated that inhibition of *de novo* FAS in naïve T cells cultured under Th17-polarizing conditions results in the downregulation of Th17-associated genes, such as *Il17a*, *Stat3*, or *Hif1a*; however, *Rorc* mRNA, coding for RORγt, remained unchanged [12]. We now tested whether the RORγt protein level is affected by blocking *de novo* FAS under Th17 polarising conditions. We found that neither inhibition nor deletion of ACC1 affected the frequencies of RORγt^+^ -expressing cells, indicating that impaired Th17 development is not merely caused by abrogated RORγt protein expression (Figure 1C).

To determine whether the expression of FoxP3 prevents RORγt^+^-expressing cells to produce IL-17 in SorA-treated cells, we made use of Scurfy × Ox40/CD30ko mice, a genetic mouse model devoid of Tregs but without lethal autoimmunity or activated T cells [39]. As expected, naïve T cells from Scurfy × Ox40/CD30ko mice could not differentiate into iTregs under iTreg-polarizing conditions (data not shown) but could develop into Th17 cells under Th17-polarizing conditions (Figure 1D). Moreover, SorA diminished IL-17 production even in the absence of FoxP3, indicating that defective FAS impairs Th17 differentiation independently from promoting FoxP3 expression.

Previous research has demonstrated that a significative fraction of intestinal CD4^+^ Treg cells simultaneously express RORγt and FoxP3 [10,11,40]. This peripheral RORγt^+^FoxP3^+^ double positive subset, which exhibits enhanced immunosuppressive functions *in vivo*, is regulated by the intestinal microbiota and represents a stable effector Treg lineage in the gut [10,11]. Given its significance *in vivo*, we sought to investigate further the role of ACC1 in the development of this subset. We analysed the expression of several Treg markers in the colonic and small intestinal lamina propria (SI), mesenteric lymph nodes (mLN), spleen, and pool of axillary and inguinal lymph nodes (pLN) derived from wild-type (WT) or TACC1 mice (Figure 1E). As previously reported, the colonic lamina propria was found to be enriched in RORγt^+^FoxP3^+^ Tregs in both groups compared to the lymphoid organs. Interestingly, TACC1 mice displayed elevated cell frequencies of RORγt^+^FoxP3^+^ double-positive cells in mLN, pLN and spleen compared to WT mice. Although not significant, the same tendency was observed in the colon but not in the small intestine (Figure 1E). These data indicate that genetic ablation of ACC1 on T cells *in vivo* may reinforce the development of RORγt^+^FoxP3^+^ Tregs, which are crucial for the suppression of inflammatory immune responses at intestinal sites. The expression of additional Treg markers, including KLRG1, Helios, IL-33 receptor (ST2), GATA3, LAG-3 and TIGIT, was evaluated (data not shown). Interestingly, TACC1 exhibited increased TIGIT^+^ Treg frequencies in the mLN, pLN and spleen, a marker of suppressive activity.

### 2.2 Inhibition of ACC1, but not ACC2, promotes Treg development

ACC1 shares significant homology with ACC2, a different isoform that controls the rate of LC-FAO [21] by blocking the activity of the carnitine palmitoyltransferase 1 (CPT1), which transports LC-FAs into the mitochondria. Both ACC1 and ACC2 enzymes have similar functional domains, including a biotin carboxylase domain, a carboxyltransferase domain, and a biotin carrier domain. Their structural homology allows them to catalyse the same biochemical reaction of converting acetyl-CoA to malonyl-CoA. Since we previously showed that LC-FAO is dispensable for Treg differentiation and function [22], we next asked whether ACC2 activity [41,42] could contribute to FAS in the absence of ACC1. To test this, we crossed ACC2 knockout mice to TACC1 mice. We first analysed ACC1-, ACC2-, and ACC1/ACC2-knockout T cells cultured under Th17-polarizing conditions. As expected (Fig. S1A), ACC1 deletion led to an increase in FoxP3 frequencies, whereas ACC2 deletion alone did not affect IL-17 production or FoxP3-expressing cells compared to WT controls (Fig. S1A). Likewise, ACC1/ACC2-double knockout did not further enhance Treg development compared to ACC1 deletion alone (Fig. S1A). Instead, SorA slightly increased FoxP3 frequencies in ACC1- and ACC1/ACC2-knockout T cells compared to cells originating from the same genetic background treated with vehicle. Next, we evaluated whether dual ACC1/ACC2 deletion alters the capacity to synthesise FAs. We cultured naïve T cells under Th17-polarising conditions in the presence of ^13^C_6_-glucose and determined the incorporation of ^13^C_6_-glucose-derived carbons into FAs. As expected, *de novo* synthesis of palmitate and stearate was impaired in TACC1 cells but not ACC2^ko^ cells. Deleting both isoforms in ACC1/ACC2-knockout T cells did not further inhibit FAS compared to ACC1 deletion alone (Fig. S1B).

Similarly, the accumulation of phospholipids and neutral lipids was reduced upon ACC1 deficiency but not further decreased by additional ACC2 deletion (Fig. S1C). Furthermore, TACC1 cells exhibited increased uptake of Bodipy-labeled palmitate compared to WT cells, yet to the same level as TACC1 × ACC2^ko^ cells (Fig. S1D). The effect of SorA on the capacity of T cells to synthesise fatty acids and lipids mimicked the impact of ACC1 deletion. Together, our results suggest that ACC1 is the only isoform of ACC responsible for *de novo* synthesis of FA in CD4^+^ T cells and involved in regulating the fate between Th17 and Treg cells. Furthermore, our results demonstrate that the effects of SorA are mediated by ACC1 [43], with no compensatory effects from ACC2.

### 2.3 Inhibition of ACC1 activity promotes the induction and stability of FoxP3

**Figure 2:**
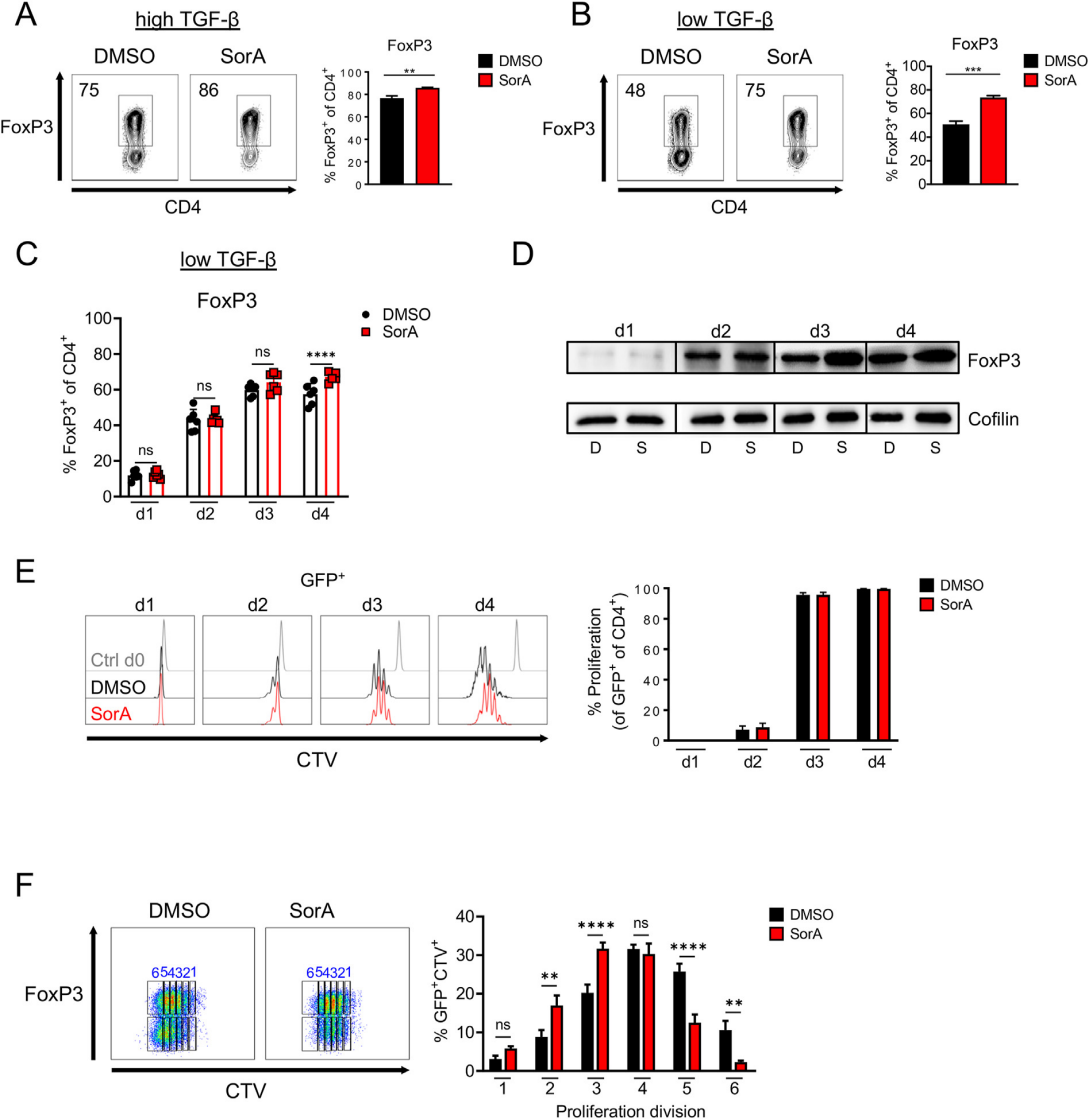
**Absence of ACC1 activity promotes Treg development in iTreg cultures.** (A–D) Naïve CD4^+^ T cells from WT mice were cultured under optimal (A) or suboptimal (B–D) iTreg-polarizing conditions in the presence of DMSO or SorA. Bar graphs display frequency of FoxP3^+^ cells among live CD4^+^ T cells on day 4 of culture (A, B) or at indicated time points (C) determined by flow cytometry. (D) FoxP3 protein was determined in whole-cell lysates by western blot. Cofilin served as a loading control. D: DMSO-treated, S: SorA-treated. (E, F) Naïve CD4^+^ T cells from DEREG mice were cultured under suboptimal iTreg-inducing conditions in the presence of DMSO or SorA and their proliferation was assessed by the cell proliferation dye CellTrace violet (CTV) over the course of the culture. (E) Bar graphs represent percentage of GFP^+^CTV^+^-proliferating cells at specified time points determined by flow cytometry. (F) Representative flow cytometry plots showing CTV^+^ and FoxP3-GFP^+^ or FoxP3-GFP^−^ cells among total live CD4^+^ T cells. Bar graphs show percentage of CTV^+^FoxP3-GFP^+^ cells in each proliferation cycle. Results are representative of two (D) or six (A, B) independent experiments or shown as pooled data from three (E, F) or six (C) independent experiments. Each symbol represents an individual experiment (C). Error bars show s.d. of pooled data (C, E, F) or triplicates (A, B). ∗∗*P* < 0.01, ∗∗∗*P* < 0.001, ∗∗∗∗*P* < 0.0001. n.s., non-significant. Two-tailed Student's t-test (A, B) or two-way ANOVA with Bonferroni correction (C, E, F). See also Figure S2. (For interpretation of the references to color in this figure legend, the reader is referred to the Web version of this article).

Next, we addressed whether the increase in FoxP3^+^ cell frequencies observed in Th17 cultures lacking ACC1 activity was simply due to the loss of FoxP3^−^ cells or a specific effect on promoting Treg development. To this aim, we cultured naïve T cells under iTreg-polarising conditions upon SorA treatment. Both pharmacological inhibition and deletion of ACC1 enhanced the generation of FoxP3^+^ Tregs (Figure 2A, Fig. S2A, B). This effect was more pronounced using suboptimal TGF-β doses for iTreg induction (Figure 2B, Fig. S2C). Furthermore, SorA-treated iTreg cultures did not exhibit increased cell death compared to the control DMSO, as evidenced by Live/Dead staining, thus negating the possibility of a preferential loss of non-Tregs (Fig. S2A).

To better understand how ACC1 inhibition promotes Treg development, we followed FoxP3 protein expression in iTregs by flow cytometry and western blot over time. FoxP3^+^ cells were detected on day 1, and their frequency (Figure 2C) and protein levels (Figure 2D) increased on day 2 of culture, showing comparable levels in the presence or absence of ACC1 activity (Figure 2C,D). At later time points, the frequency and protein levels of FoxP3 were higher in SorA-treated iTregs, in contrast to those treated with the vehicle control.

To exclude the possibility that the increase in FoxP3^+^ T cell frequencies observed throughout the culture was due to an outgrowth of FoxP3^-^ cells in the culture, the proliferation rate of iTregs generated from naïve T cells of DEREG mice [3] with or without SorA was assessed over time under suboptimal iTreg culture conditions by CellTrace Violet (CTV) labelling. Although SorA did not alter the overall proliferation rate of GFP^+^FoxP3^+^ and GFP^−^FoxP3^−^ cells (Figure 2E, Fig. S2D), it equally arrested the proliferation division of both FoxP3^+^GFP^+^ and FoxP3^−^GFP^−^ cell fractions from round five of division onwards compared to the vehicle control (Figure 2F, Fig. S2E). Therefore, our findings indicate that the observed increase in FoxP3^+^ frequencies following ACC1 inhibition is not attributable to a FoxP3^−^ cell expansion.

**Figure 3:**
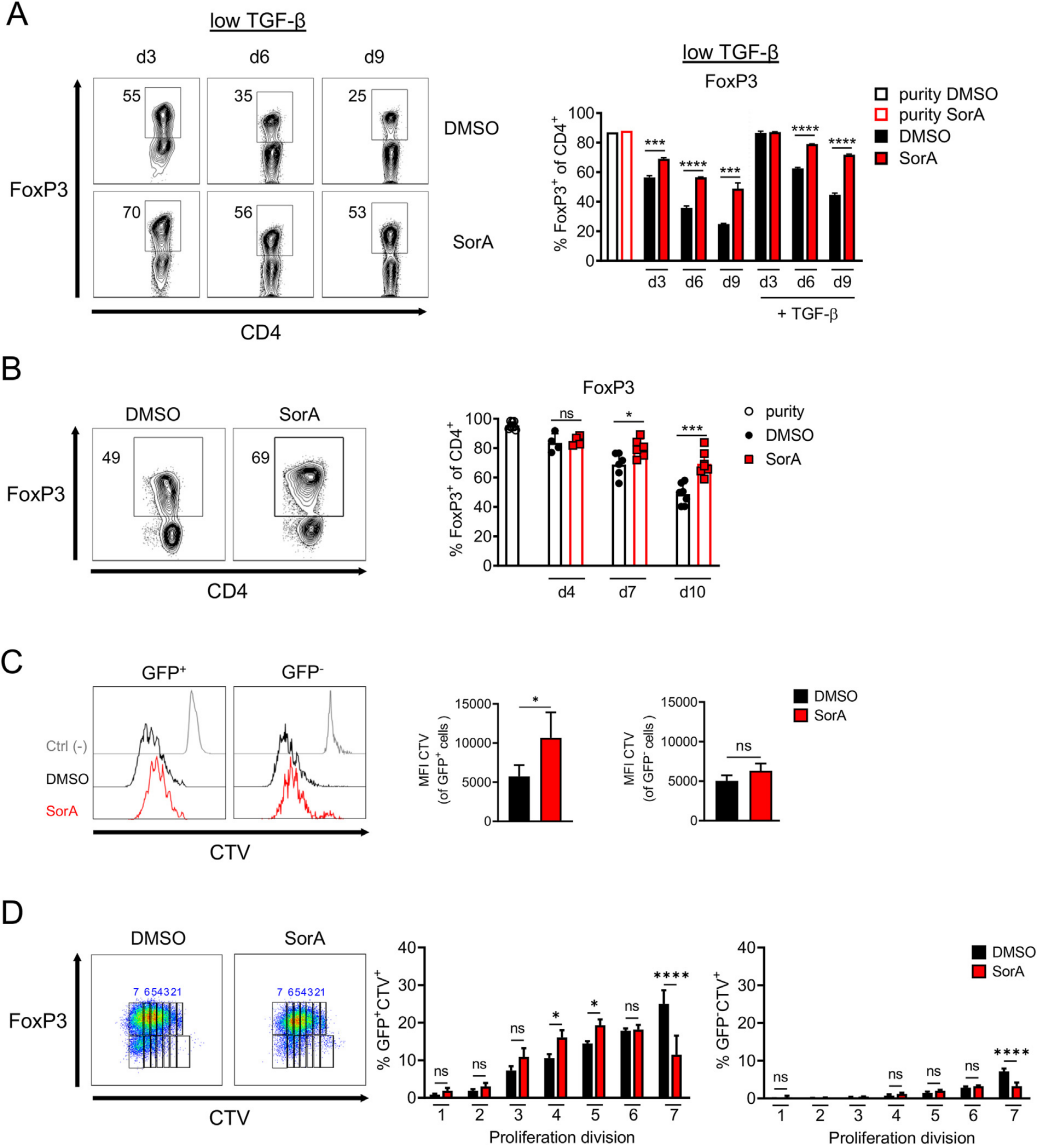
**Absence of ACC1 activity promotes Treg stability.** (A) Naïve CD4^+^ T cells from DEREG mice were cultured under suboptimal iTreg-inducing conditions in the presence of DMSO or SorA. After differentiation, GFP^+^FoxP3^+^ iTregs were re-sorted and plated in 200 U/ml IL-2 with or without TGF-β (1 ng/mL). FoxP3 frequency in live CD4^+^ T cells was determined directly after re-sort (purity) and at different time points after re-plating by flow cytometry. Left, Representative flow cytometry showing FoxP3 expression in live CD4^+^ T cells. Right, bar graphs display frequency of FoxP3^+^ cells among live CD4^+^ T cells. (B–D) CD4^+^GFP^+^FoxP3^+^ nTregs were sorted from DEREG mice and expanded *ex vivo* with CD3ε/CD28 stimulation in the presence of DMSO or SorA. (B) From day 4 onwards, cells were cultured in absence of CD3ε/CD28 stimulation. At different time points, remaining FoxP3 protein expression in live CD4^+^ T cells was determined by flow cytometry. (C, D) Sorted CD4^+^GFP^+^FoxP3^+^ nTregs were labelled with CTV and their proliferation was assessed by flow cytometry on day 4. (C) Bar graphs shows the MFI of CTV among live CD4^+^GFP^+^FoxP3^+^ or CD4^+^GFP^−^FoxP3^-^ cells. (D) Representative flow cytometry plots showing the percentage of CTV^+^ and GFP^+^FoxP3^+^ or GFP^−^FoxP3^-^ cells among total live CD4^+^ T cells. Bar graphs show percentage of CTV^+^GFP^+^FoxP3^+^ or CTV^+^GFP^+^FoxP3^-^ cells in each proliferation cycle. Results are representative of three (A) independent experiments or shown as pooled data from three (C, D) or four to eight (B) independent experiments. Each symbol represents an individual experiment. Error bars show s.d. of pooled data (B–D) or triplicates (A). ∗*P* < 0.05, ∗∗∗*P* < 0.001, ∗∗∗∗*P* < 0.0001. n.s., non-significant. Two-way ANOVA with Bonferroni correction (A, B, D) or Two-tailed Student's t-test (C). See also Figure S3.

Next, we addressed the effect of SorA on Treg stability. GFP^+^FoxP3^+^ iTregs generated from naïve T cells of DEREG mice were cultured with or without SorA under suboptimal (Figure 3A) or optimal iTreg culture conditions (Fig. S3A) and re-sorted after differentiation for high purity by FoxP3-GFP marker expression. Sorted cells were cultured in the presence of IL-2, with or without TGF-β, for up to 9 days, and FoxP3 protein expression was assessed at different time points by flow cytometry. Importantly, SorA was only present in the initial differentiation phase but not during the stability assay. We observed that FoxP3 protein expression declined over time, but previously blocking ACC1 activity during the initial iTreg differentiation phase reduced the loss of FoxP3 expression, both in iTregs generated under suboptimal (Figure 3A) or optimal conditions (Fig. S3A). TGF-β supplementation slowed down the continuous loss of FoxP3, but also, under these conditions, FoxP3 frequencies remained higher in SorA-differentiated iTregs than in vehicle-differentiated iTregs (Figure 3A, Fig. S3A).

We then evaluated the effect of ACC1 inhibition on nTreg stability. To this aim, CD4^+^GFP^+^FoxP3^+^ T cells from DEREG mice were isolated and cultured *ex vivo* in the presence of SorA or vehicle control for four days, and FoxP3 protein expression was analysed over time. From day 7 onwards, SorA also improved the stability of *ex vivo*-expanded FoxP3^+^ nTregs (Figure 3B, Fig. S3B). Finally, *ex-vivo* isolated CD4^+^GFP^+^FoxP3^+^ nTreg were labelled with CTV and expanded in the presence of SorA or vehicle control for 4 days. The proliferation was examined by flow cytometry. Although SorA still had no apparent effect on nTreg stability after 4 days of culture compared to the control condition (Figure 3B), it dramatically dampened the proliferation of GFP^+^Foxp3^+^ nTregs (Figure 3C), as observed in the final round of cell division for both GFP^+^CTV^+^ and GFP^−^ CTV^+^ cell fractions, most likely due to fatty acid deprivation (Figure 3C,D). Interestingly, the frequency of GFP^−^CTV^+^ cells was significantly lower in each round of proliferation in both conditions compared to its counterpart, indicating that Treg expansion was favoured over the expansion of the non-Treg fraction for both conditions (Figure 3D). Together, these results suggest that the lack of ACC1 activity promotes not only Treg development but also stability and that both effects are not simply due to the loss of non-Treg cells in the culture.

### 2.4 ACC1 inhibition elevates intracellular acetyl-CoA levels

**Figure 4:**
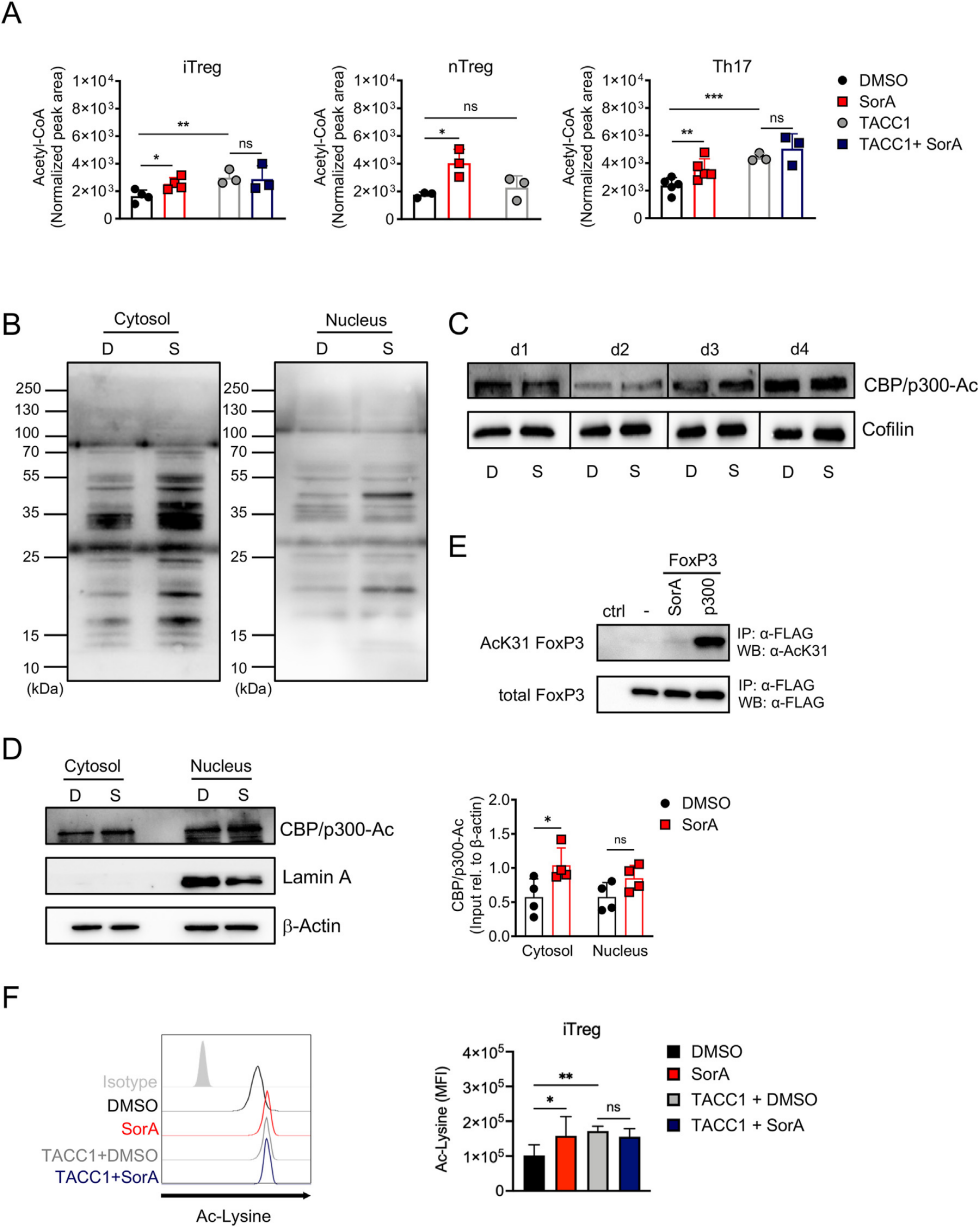
**ACC1 inhibition increases intracellular acetyl-CoA and protein acetylation levels.** (A) Intracellular relative concentration of acetyl-CoA was determined in iTreg and Th17 cultures after two days and in *ex-vivo*-expanded nTregs after 4 days by mass spectrometry. Graphs show acetyl-CoA peak area counts normalised to total protein. (B–D) Naïve CD4^+^ T cells from WT mice were cultured under suboptimal iTreg-polarizing conditions in the presence of DMSO or SorA. Acetylated-Lysine (B) and acetylated-CBP/p300 (D) proteins were determined in cytoplasmic and nuclear lysates (B, D) or in whole-cell lysates (C) by western blot on day 4. Cofilin (C) or β-actin (D) served as a loading control while Lamin A (D) as a nuclear extraction control. D: DMSO-treated, S: SorA-treated. (D) Bar graphs show fold induction of acetylated-CBP/p300 normalised to β-Actin. (E) 293T cells were transfected with FLAG-FoxP3 and/or HA-p300 expression vectors and treated for 16 h with SorA (1 μM). H_2_O served as a negative control. FLAG-FoxP3 was immunoprecipitated with anti-FLAG agarose followed by western blot using anti-AcK31 FoxP3 and anti-FLAG antibodies. (F) Naïve CD4^+^ T cells from WT or TACC1 mice were cultured under suboptimal iTreg-polarizing conditions in the presence of DMSO or SorA for 4 days and the expression of acetylated-Lysine among CD4^+^FoxP3^+^ cells was detected by flow cytometry. Bar graphs display the MFI of Acetylated-Lysine among live CD4^+^FoxP3^+^ cells on day 4. Results are representative of three (B–C, E) independent experiments or shown as pooled data from three to six (A), four (D) or six (F) independent experiments. Each symbol represents an individual experiment (A, D). Error bars show s.d. ∗*P* < 0.05, ∗∗*P* < 0.01, ∗∗∗*P* < 0.001. n.s., non-significant. One-way ANOVA (A, F) or Two-way ANOVA with Bonferroni correction (D).

Next, we aimed to dissect how ACC1 deficiency promotes and stabilises FoxP3 levels. Previous studies using non-immune systems, such as yeast [25], cancer cells [23], and hepatocytes [24], have demonstrated that the absence of ACC1 activity results in increased histone and global protein acetylation associated with elevated intracellular acetyl-CoA levels [23]. Thus, we hypothesised that the lack of ACC1 activity in T cells required to convert acetyl-CoA to malonyl-CoA would lead to the accumulation of intracellular acetyl-CoA. In line with this, we had previously observed that malonyl-CoA was utterly absent in SorA-treated cells [12]. We determined intracellular acetyl-CoA concentrations in *ex vivo*-expanded nTregs and naïve T cells derived from WT or TACC1 mice cultured under iTreg- or Th17-polarizing conditions in the presence or absence of SorA via mass spectrometry. Indeed, higher levels of acetyl-CoA were detected upon SorA treatment in nTregs, iTregs and Th17 cells (Figure 4A). Since SorA can block not only ACC1 but also ACC2 [[44], [45], [46]], and to exclude a possible unspecific effect due to residual ACC1 activity [47], we included SorA-treated iTregs or Th17 cells from TACC1 mice in our analysis. Remarkably, comparable levels of acetyl-CoA were observed between TACC1-derived iTregs or Th17 treated with vehicle and SorA.

Considering the increased intracellular acetyl-CoA concentrations found upon FAS inhibition, we reasoned that ACC1 inhibition may lead to elevated protein acetylation. We therefore examined total levels of lysine acetylation in the cytosol and nucleus from iTregs. Interestingly, ACC1 inhibition dramatically altered the acetylation pattern of a wide range of proteins not only in the cytosol but also in the nucleus of iTregs (Figure 4B), suggesting that the intracellular elevation of acetyl-CoA caused by SorA can indeed drive increased cytosolic and nuclear protein acetylation.

Then, we questioned whether SorA stabilises FoxP3 protein by directly increasing its acetylation, thereby preventing its degradation [35,36]. To address this, first, we assessed the expression levels of acetylated CBP (lysine 1535)/p300 (lysine 1499), a HAT indispensable for Treg cell development and function [48] (Figure 4C,D). Whole-cell lysates obtained from iTregs treated with SorA or vehicle control were analysed by western blot over time. Notably, even though acetylated CBP/p300 increased over time, SorA treatment did not influence its acetylation compared to the control (Figure 4C). To further test whether SorA treatment differentially affects the cytosolic and nuclear acetylation of CBP/p300, we isolated both protein fractions from iTregs after four days of differentiation. As expected, acetylated CBP/p300 was found in the cytosol and nucleus in both conditions. Interestingly, SorA-treated iTreg showed significantly higher levels of acetylated CBP/p300 in the cytosolic fraction (Figure 4D).

Since the detection of distinct FoxP3 protein acetylation sites in primary Tregs is technically challenging, we overexpressed FoxP3 in 293T cells, immunoprecipitated FoxP3, and determined FoxP3 protein acetylation using an acetylation-specific FoxP3 antibody (AcK31 FoxP3 [35]) by western blot. As reported previously [35], co-transfection with the HAT p300 resulted in strong FoxP3 protein acetylation (Figure 4E). However, in this system, SorA treatment did not substantially increase FoxP3 protein acetylation compared to the untreated control group (Figure 4E). We next detected total acetyl-lysine levels in iTreg cultures over time by flow cytometry. SorA treated-FoxP3^+^ iTregs displayed significantly enhanced levels of acetylated-lysine after 4 days of differentiation compared to the vehicle control (Figure 4F). Similarly, ACC1-deficient FoxP3^+^ iTregs exhibited markedly increased lysine acetylation on day 4, when compared to the control group. Similarly to what was observed for acetyl-CoA concentrations, comparable lysine acetylation was found in SorA-treated or untreated ACC1-deficient iTregs (Figure 4F).

A dynamic switch of acetylation and ubiquitination regulates FoxP3 protein stability. Both post-translational modifications compete for identical lysine residues, where acetylation prevents FoxP3 ubiquitination and proteasomal degradation [35,36]. Ubiquitination is a reversible process in which ligases catalyse the addition of ubiquitin, whereas deubiquitinating enzymes (DUBs) catalyse the opposite reaction [49] (Fig. S4A). The DUB USP7 is critical for Treg maintenance since inhibition or deletion of USP7 results in decreased FoxP3 protein stability and augmented anti-tumour immunity [50,51] (Fig. S4A). Here, we made use of this reciprocal regulation of FoxP3 stability to address whether ACC1 inhibition, potentially inducing FoxP3 protein acetylation and pre-occupying the lysine binding sites, would prevent ubiquitin-mediated degradation of FoxP3 upon USP7 inhibition (Fig. S4A). We expanded nTregs *ex vivo* or generated iTregs with or without SorA. After 4 days, where Treg stability is still comparable between vehicle- and SorA-treated nTregs (Figure 3B, Fig. S4D), cells were treated with the USP7 inhibitor (DUBI) P5091 for 5 h (Fig. S4B). Of note, the effect of protein ubiquitination induced by USP7 inhibition was slightly reduced in samples pre-treated with SorA both in nTregs (Fig. S4C) and iTregs (Fig. S4E). However, in both Treg cell types, pre-treatment with SorA did not influence ubiquitin-mediated loss of FoxP3 protein induced by USP7 inhibition (Figure S4D, F).

In summary, while we could not directly detect increased acetylation of the FoxP3 protein, ACC1 inhibition significantly raises intracellular acetyl-CoA levels, promoting overall protein acetylation during Treg differentiation. Notably, FoxP3^+^ cells treated with SorA or derived from TACC1 mice exhibit markedly higher levels of lysine acetylation, further supporting the role of ACC1 inhibition in enhancing Treg differentiation and maintenance through widespread protein acetylation. These findings highlight the critical link between metabolic shifts and the development and stabilisation of FoxP3^+^ Tregs.

### 2.5 Blocking ACC1 enhances *Foxp3* transcription

**Figure 5:**
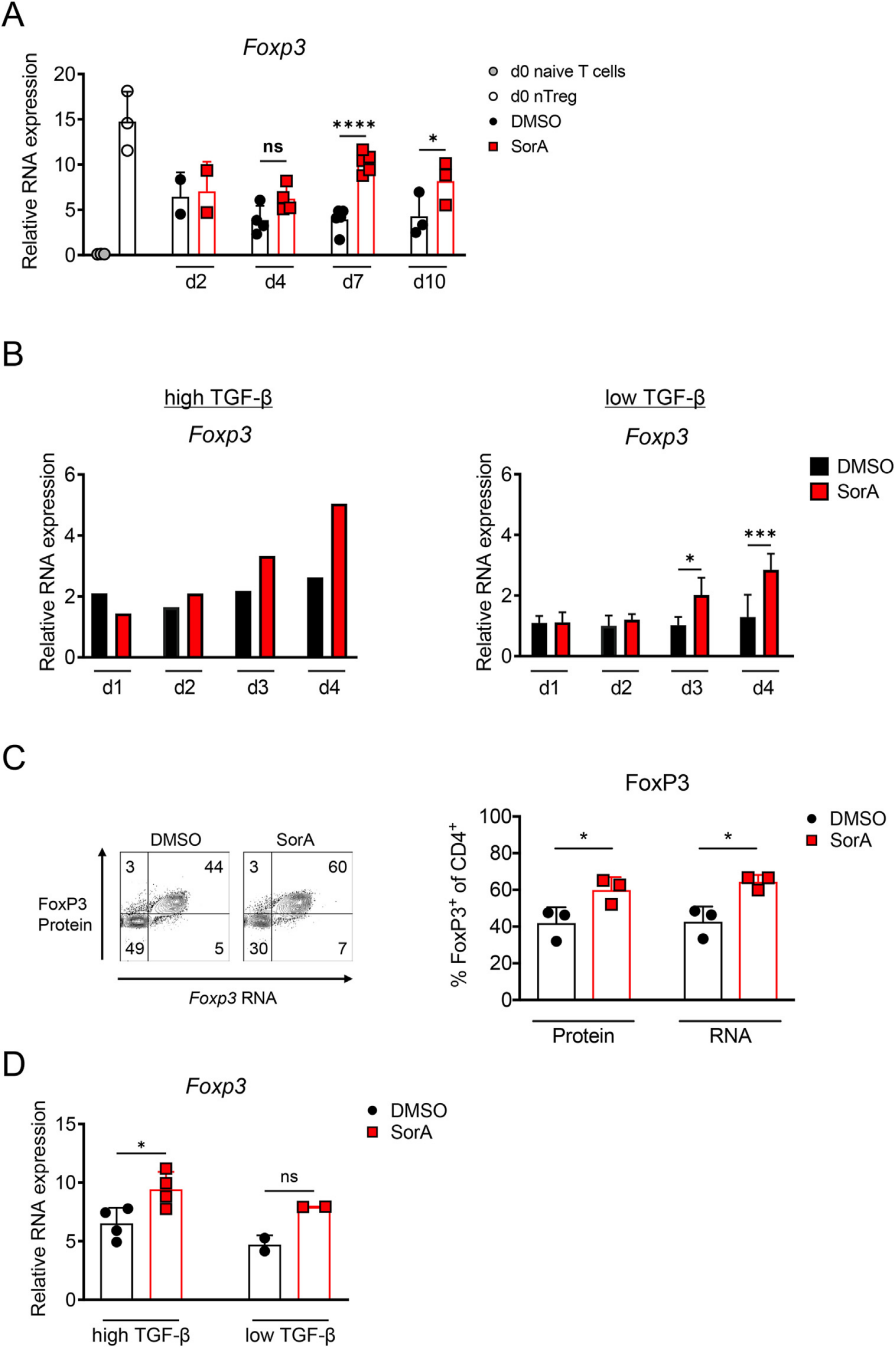
**Blocking ACC1 activity promotes *Foxp3* mRNA expression.** (A–B) *Foxp3* mRNA expression was analysed by RT-PCR in *ex vivo*-expanded nTregs (A) and naïve T cells cultured under optimal or suboptimal iTreg-polarizing conditions (B) in the presence of DMSO or SorA. Cycle threshold values were normalised to *Actb,* and the relative expression ×10^3^ is displayed. (C) Naïve T cells were co-stimulated with CD3ε/CD28 mAb-coated beads, treated with TGF-β (3 ng/mL) and IL-2 (25 U/mL) in the presence of DMSO or SorA and PrimeFlow analysis was performed on day 4. Representative flow cytometric plots of FoxP3 protein and RNA expression. Graphs show the frequency of FoxP3-protein^+^ or *Foxp3*-RNA^+^ cells among live CD4^+^ T cells determined by flow cytometry. (D) Naïve T cells from DEREG mice were cultured under optimal or suboptimal iTreg-inducing conditions in the presence of DMSO or SorA. On day 4, GFP^+^FoxP3^+^ iTregs were re-sorted and *Foxp3* mRNA expression was determined by RT-PCR. Cycle threshold values were normalised to *Actb,* and relative expression ×10^3^ is displayed. Results are representative of two (B: optimal) independent experiments or shown as pooled data from two to five (A), three (B: suboptimal, C), or two to four (D) independent experiments. Each symbol represents an individual experiment. Error bars show s.d. ∗*P* < 0.05, ∗∗∗∗*P* < 0.0001, ∗∗∗*P* < 0.001. n.s., non-significant. One-way ANOVA (C, D) or Two-way ANOVA (A, B: suboptimal) with Bonferroni correction. See also Figure S5.

A sustained Treg phenotype is not only dependent on FoxP3 protein stability but also on a continuous transcription of *Foxp3* [52]. Thus, we next determined the effect of ACC1 inhibition on *Foxp3* gene expression. SorA maintained higher *Foxp3* mRNA levels in *ex vivo*-expanded nTregs from day 7 onwards (Figure 5A) and increased *Foxp3* mRNA during iTreg formation under optimal or suboptimal conditions (Figure 5B). Using PrimeFlow experiments, which facilitates to simultaneously measure RNA and protein expression by flow cytometry in the same cells, we observed that iTregs cultured with SorA showed not only elevated FoxP3 protein expression but also increased *Foxp3* mRNA expression compared to vehicle-treated iTregs (Figure 5C). Consequently, we also analysed *Foxp3* mRNA expression in re-sorted GFP^+^FoxP3^+^ cells generated under optimal or suboptimal iTreg culture conditions from naïve CD4^+^ T cells of DEREG mice with or without SorA. This experimental system allowed us to discriminate the effect of ACC1 inhibition on *Foxp3* transcription without a potential bias due to differences in FoxP3 protein levels during Treg formation. Strikingly, re-sorted GFP^+^FoxP3^+^ iTregs generated in the presence of SorA exhibited higher *Foxp3* mRNA expression compared to iTregs generated in the presence of a vehicle control (Figure 5D), despite equal FoxP3 frequencies in both groups (Fig. S5A). These results suggest that the increase in Treg formation and stability upon ACC1 inhibition is driven by enhanced transcriptional activity of the *Foxp3* gene locus.

### 2.6 ACC1 inhibition promotes Treg development and stability by enhanced histone acetylation

**Figure 6:**
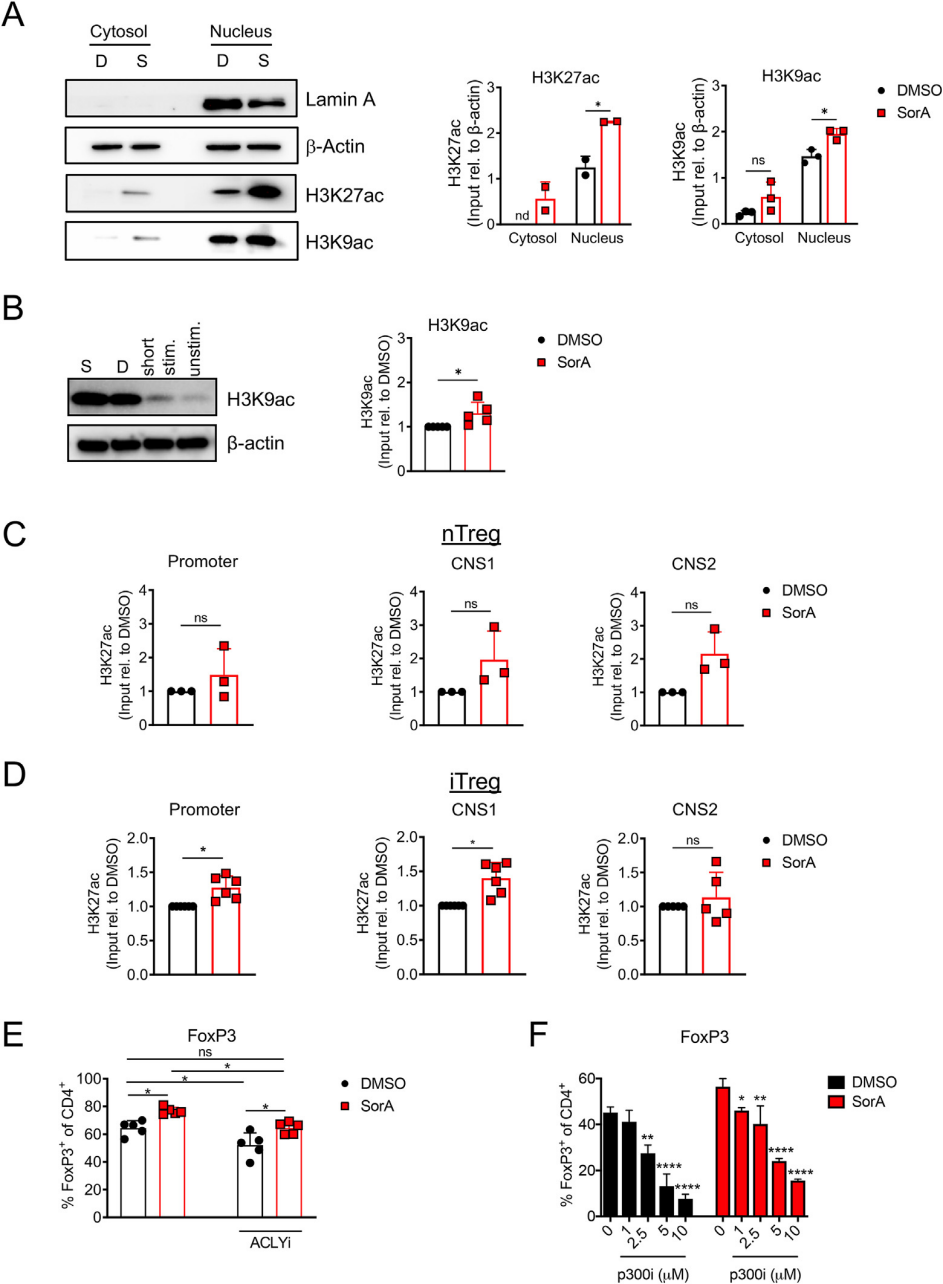
**ACC1 inhibition increases chromatin accessibility within the *Foxp3* locus.** (A) Naïve CD4^+^ T cells from WT mice were cultured under suboptimal iTreg-polarizing conditions in the presence of DMSO or SorA. Acetylated H3K27 and H3K9 proteins were determined in cytoplasmic and nuclear lysates by western blot on day 4. β-actin and Lamin A served as a loading or nuclear extraction control, respectively. D: DMSO-treated, S: SorA-treated. Bar graphs show fold induction of H3K27ac and H3K9ac normalised to β-Actin. n.d., not detected. (B) Naïve CD4^+^ T cells were co-stimulated with CD3ε/CD28 mAb-coated beads in the presence of DMSO or SorA for 16 h. Whole-cell lysates were analysed by western blot using an acetyl-H3K9 antibody. Unstimulated and shortly CD3/CD28-activated naïve CD4^+^ T cells served as controls to determine baseline acetylation of H3K9. Bar graphs show fold induction of H3K9 acetylation normalised to DMSO treatment after normalisation to β-Actin. (C–D) nTregs were expanded *ex vivo* (C) and naïve CD4^+^ T cells were cultured under suboptimal iTreg-inducing conditions (D) in the presence of DMSO or SorA. Cells were harvested after four days (C) or 62 h (D) and subjected to H3K27 acetylation ChIP. Graphs show ChIP analysis of H3K27 acetylation at *Foxp3* locus in nTregs (C) and iTregs (D). Values are presented as input relative to DMSO, after normalisation to input DNA. (E, F) Naïve CD4^+^ T cells were cultured under suboptimal iTreg-polarizing conditions in the presence of DMSO or SorA and 10 μM of the ACLY inhibitor BMS 303141 (E) or indicated concentrations of the p300 inhibitor C646 (F). Graphs show the frequency of FoxP3^+^ cells among live CD4^+^ T cells determined by flow cytometry on day 4. Results are representative of four independent experiments (F) or shown as pooled data from two to three (A), three (C), five (B, D: CNS2, E) or six (D) independent experiments. Each symbol represents an individual experiment. Error bars show s.d. of triplicates (E) or pooled data (A–C, D) ∗*P* < 0.05. n.s., non-significant. Two-tailed Student's t-test (B) or two-tailed paired Wilcoxon signed-rank test (C, D) or one-way ANOVA with Bonferroni correction (E, F). See also Figure S5.

Histone acetylation opens the chromatin, allowing the binding of polymerases and transcription factors that enhance gene transcription. In particular, acetylated lysine K9 and K27 of histone H3 (H3K9 and H3K27) have been associated with active promoters and initiation of *Foxp3* gene transcription [31,53,54]. As previously shown for total H3 acetylation in non-immune yeast [25] and cancer cells [23], ACC1 inhibition not only increased nuclear H3K27 and H3K9 acetylation in iTregs after 4 days of differentiation but also increased H3K9 acetylation in TCR-activated naïve T cells (Figure 6A,B).

To substantiate these findings, we generated assay for transposase-accessible chromatin following sequencing (ATAC-seq) and histone H3K27-acetylation (ac) chromatin immunoprecipitation sequencing (ChIP-seq) data sets from iTreg cells derived from DEREG and TACC1 mice treated with vehicle or SorA 62 h after the start of culture. Principal component analysis (PCA) (Fig. S6A) and genome tracks of Treg-specific example loci (*Ctla4, Il2ra*) (Fig. S6B) obtained from ATAC-seq revealed that SorA has no significant impact on the chromatin landscape in TACC1-iTregs, thereby supporting our hypothesis that SorA effects on Treg development are specifically mediated via ACC1. Most importantly, TACC1-iTregs exhibited increased H3K27ac signals (Fig. S6C) as exemplified in the *Rorc* locus and the Treg-specific loci *Il2ra* and *Ctla4* (Fig. S6B) in comparison to vehicle-treated iTregs derived from DEREG mice. Indeed, TACC1-iTregs showed 3499 induced H3K27ac peaks and 3295 differentially induced accessible peaks (Fig. S6D) compared to vehicle-treated iTregs derived from DEREG mice. In contrast to the complete ACC1 knockout, SorA-treated iTregs from DEREG mice showed only a slight increase in chromatin accessibility compared to vehicle treatment (Figure S6C,E).

After demonstrating that the absence of ACC1 deletion increases chromatin accessibility on a global level, we determined histone acetylation specifically within the *Foxp3* locus, including the promoter, CNS1, and CNS2 regions, of *ex vivo*-expanded nTregs after 4 days as well as of naïve T cells polarised under iTreg-inducing conditions after 62 h, respectively. ChIP qPCR analysis using H3K27 acetylation as a permissive histone mark revealed that SorA induced hyperacetylation in the *Foxp3* gene locus of iTregs and nTregs, yet with a straightforward but not significant tendency in nTregs (Figure 6C,D). As expected, iTregs showed the strongest acetylation in the TGF-β-inducible CNS1 region (Fig. S5B). At the time points investigated, control- and SorA-treated nTreg (day 4; Figure 3B) and iTreg (62 h; Fig. S5C) cultures yet exhibited comparable FoxP3 expression, arguing against a bias toward stronger acetylation within the *Foxp3* locus due to overall higher FoxP3 levels in SorA-treated samples.

The requirements for histone acetylation are the activity of HATs and the availability of their substrate acetyl-CoA. For HATs, the activity of p300 is critical for Treg development and homeostasis [48]. Concerning substrate availability, the nucleo-cytosolic enzyme ATP citrate lyase (ACLY), generating acetyl-CoA from citrate, was described as the primary source of acetyl-CoA for histone acetylation [55]. To address whether the Treg-promoting and stabilising effects of ACC1 inhibition are dependent on histone acetylation, we cultured naïve T cells under iTreg-polarizing conditions in the presence of BMS 303141 (Figure 6E), an inhibitor of ACLY, or in the presence of C646 (Figure 6F), an inhibitor of p300, respectively. In both approaches, limiting histone acetylation negated the FoxP3-inducing effect of SorA and further decreased FoxP3 frequencies in DMSO controls.

Together, our data suggest that the effect of ACC1 inhibition on Treg development and stability is due to the increased availability of acetyl-CoA, which enables histone acetylation within the *Foxp3* locus and facilitates enhanced and sustained *Foxp3* gene expression and, consequently, protein levels.

### 2.7 Transfer of SorA-primed iTregs ameliorates inflammatory diseases *in vivo*

**Figure 7:**
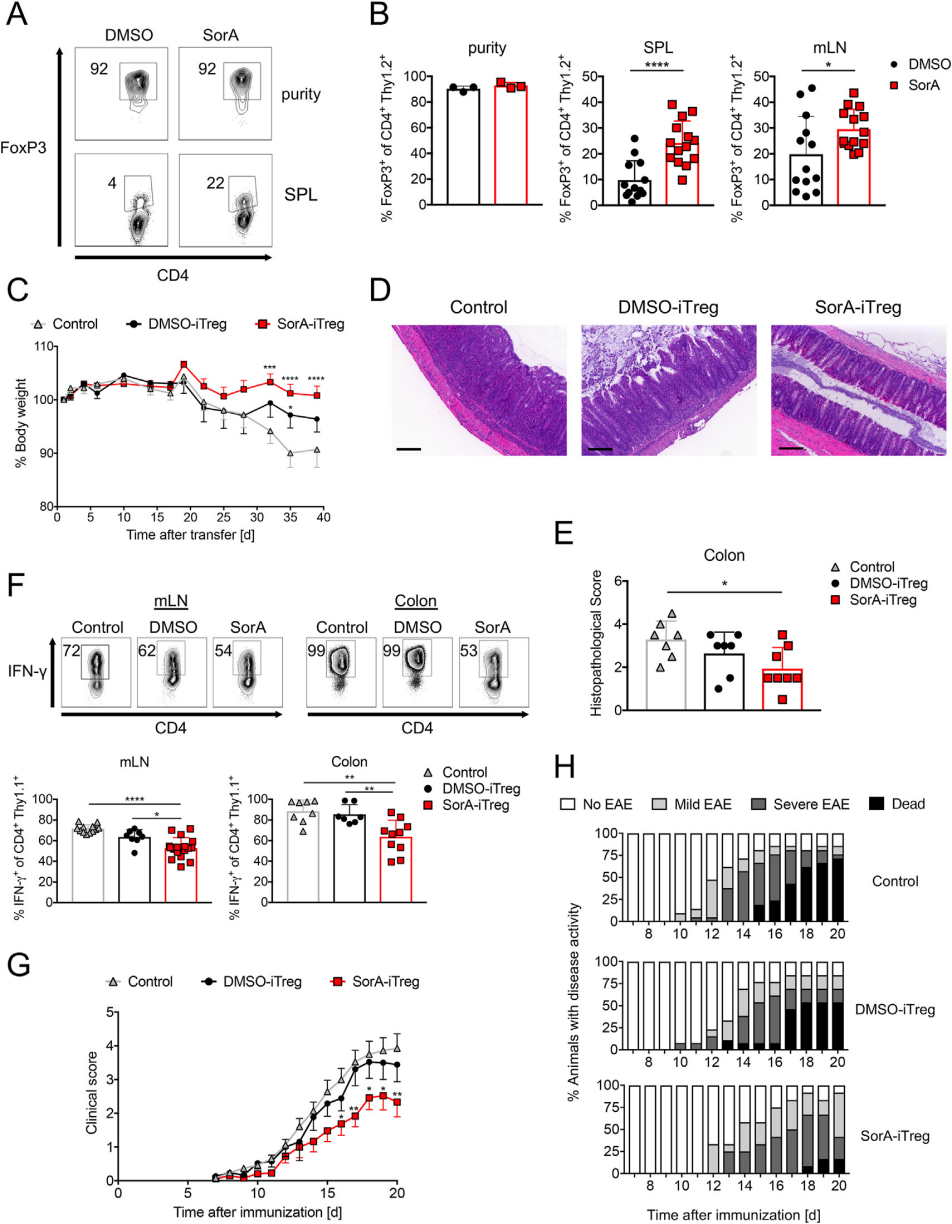
**Transfer of SorA-primed Tregs ameliorates inflammatory diseases *in vivo*.** Naïve T cells from DEREG mice were cultured under optimal iTreg-inducing conditions in the presence of DMSO or SorA, and GFP^+^FoxP3^+^ iTregs were re-sorted on day 4. (A–F) Naïve T cells (CD4^+^CD25^−^CD45RB^hi^CD90.1^+^) from Thy1.1 mice were transferred alone or together with CD90.2^+^ DMSO-iTregs or SorA-iTregs into Rag2^ko^ mice. Mice were analysed 6–7 weeks after the cell transfer. (A) Representative flow cytometry plots showing FoxP3 protein expression in iTreg cells before and after transfer. (B) Frequency of FoxP3^+^ cells among live CD4^+^CD90.2^+^ T cells before and after transfer in spleen and mLN. (C) The body weight curve of recipient mice presented as percentage of initial weight. (D) Representative H&E stainings of colon sections. Scale bar represents 200 μm. (E) Quantification of histological score. (F) Top, representative flow cytometry plots showing IFN-γ production of CD4^+^CD90.1^+^ T cells in mLN and colon. Bottom, frequency of live IFN-γ-producing CD4^+^CD90.1^+^ cells in mLN and colon determined by flow cytometry. Gating strategy is shown in Figure S7A (G, H) C57Bl/6 mice were immunised with MOG_35-55_ in complete Freund's adjuvant and pertussis toxin to induce EAE and GFP^+^FoxP3^+^ iTregs generated in presence or absence of SorA were transferred on day 3. (G) Graph shows the EAE clinical score. (H) Distribution of disease severity and mortality. On a scale of 0–5: No EAE = score <1; Mild EAE = score 1–2; Severe EAE = score 2.5–4; Dead = score 5. Results are pooled from two (D, E, F: colon, G, H) or three (A, B, C, F: mLN) independent experiments with n = 3–7 (A–F) or 6–11 (G, H) mice per group. Each symbol represents an individual mouse. Error bars show s.d. (A, B, D–F) or s.e.m. (C, G). ∗*P* < 0.05, ∗∗*P* < 0.01, ∗∗∗∗*P* < 0.001. n.s., non-significant. Two-tailed Student's t-test (B: DMSO versus SorA), one-way ANOVA with Bonferroni correction (E, F), or two-way ANOVA with Bonferroni correction (C, G). See also Figure S7.

We have recently demonstrated that the genetic ablation of ACC1 in T cells ameliorates skin inflammation in an experimental model of psoriasis by restraining the T cell effector immune response in skin lesions and increasing the frequency of effector Tregs in skin-draining lymph nodes (LNs) [56]. The stabilising effect of ACC1 inhibition on FoxP3 expression *in vitro* prompted us to test the capacity of SorA-primed Tregs to suppress inflammatory diseases. Using the adoptive transfer colitis model, we first determined the long-term stability of SorA-primed iTregs *in vivo*. We transferred CD4^+^CD25^−^CD45RB^hi^ naïve T cells from Thy1.1 mice together with re-sorted Thy1.2^+^ GFP^+^FoxP3^+^ iTregs, generated from naïve cells of DEREG mice in the presence or absence of SorA, into Rag2^−/−^ mice. After 6–7 weeks, we checked for the presence of transferred Thy1.2^+^ iTregs in spleen and mesenteric lymph nodes (mLN) of recipient mice and determined the remaining FoxP3 expression (Figure 7A,B). DMSO-primed iTregs showed drastically reduced FoxP3 frequencies with only 10% in spleen and 20% in mLN, respectively, whereas SorA-primed iTregs maintained FoxP3 frequencies at around 30% in both spleen and mLN.

Control mice gradually lost weight (Figure 7C), and the histological analysis showed loss of the typical crypt structure and intense goblet cell depletion, together with apparent inflammatory cell infiltration (Figure 7D,E). While the transfer of DMSO-primed iTregs led to an intermediary phenotype, SorA-primed iTregs markedly reduced weight loss (Figure 7C) and intestinal inflammation (Figure 7D,E). This reduction in inflammation and pathology was associated with reduced frequencies of IFN-γ-producing CD90.1^+^ T effector cells in mLN and colon (Figure 7F, Fig. S7A). Of note, in mice co-transferred with SorA-iTregs, CD90.1^+^ T effector cells secreted higher levels of IL-17A (Figure S7A,B).

To conclusively demonstrate that adoptively transferred SorA-primed iTregs are superior to vehicle-primed iTregs in controlling inflammatory responses, we used a different model of Th1/Th17 driven inflammatory disease, the experimental autoimmune encephalomyelitis (EAE) mouse model of human multiple sclerosis disease. In contrast to DMSO-primed iTregs, adoptively transferred SorA-primed iTregs ameliorated the severity and mortality of EAE (Figure 7G,H). Our data show that inhibiting ACC1 activity during iTreg differentiation improves their long-term stability and capacity to ameliorate inflammatory diseases *in vivo*.

## 3 Discussion

Over the past decade, various studies have revealed key links between immune cell functions and intrinsic metabolic pathways. Our earlier research showed that blocking or deleting ACC1 in T cells cultured under Th17-promoting conditions restrains Th17 differentiation and promotes Treg development [12]. This study further investigates the molecular mechanisms underlying this Treg-promoting effect and evaluates ACC1 as a target for generating induced Tregs (iTregs) for therapeutic treatment.

Our findings demonstrate that inhibiting ACC1 during Th17 differentiation does not affect RORγt, the main transcription factor for Th17 differentiation, but rather promotes the accumulation of double-positive RORγt^+^FoxP3^+^ cells both *in vitro* and *in vivo*. Double positive RORγt^+^FoxP3^+^ cells are vital in maintaining immune balance in the gut, where constant exposure to commensal microbes and dietary antigens occurs [10,11,[57], [58], [59]]. Given that SorA is a microbial metabolite, it is plausible that other gut-derived metabolites may modulate ACC1 activity to promote RORγt^+^FoxP3^+^ cells, contributing to immune tolerance toward commensal organisms.

The two ACC isoforms, ACC1 and ACC2, serve different roles: ACC1 regulates cytosolic fatty acid synthesis (FAS), and ACC2 mitochondrial fatty acid oxidation. To determine whether ACC2 contributes to the effects of SorA on Treg development, we created double knockout (DKO) mice by crossing ACC1-knockout (TACC1) mice with ACC2 knockout mice. Our results indicate that, in contrast to ACC1, ACC2 deletion does not significantly impact Treg formation. These findings underscore that while ACC1 is critical for Treg differentiation, ACC2 is dispensable in this context, aligning with our previous research showing that Cpt1a and mitochondrial long-chain fatty acid oxidation do not regulate Treg development [22,60]. Consistent with these findings, ATAC-seq and ChIP-seq analysis confirm that SorA's effects on Treg development are specific to ACC1.

Beyond promoting Treg differentiation, ACC1 inhibition also maintains FoxP3 expression across multiple contexts, including Th17 cultures, *in vitro*-expanded iTregs, and *ex vivo*-expanded natural Tregs (nTregs). Notably, the presence of SorA merely in the differentiation phase of iTregs was sufficient to promote their long-term stability.

Treg stability is regulated on different levels: TSDR demethylation and histone acetylation at the *Foxp3* locus and direct acetylation of the FoxP3 protein itself [[61], [62], [63]]. Acetyl-CoA availability is a critical regulator of these processes as it serves as a substrate for lysine acetyltransferases (KATs) [64]. Our data shows that inhibition of ACC1 elevates acetyl-CoA levels promoting global protein acetylation and reducing ubiquitination.

Although previous studies have demonstrated that FoxP3 protein acetylation prevents its degradation via the ubiquitin-proteasome system, we did not detect substantial FoxP3 acetylation using a modification-specific antibody against acetylated K31 in FoxP3 [35,36]. This could be due to limitations in our experimental model, where overexpression of FoxP3 in transfected cells may obscure acetylation effects. Similarly, we could not detect changes in ubiquitin-mediated degradation of FoxP3 protein after SorA treatment in iTregs or nTregs. Additional factors might be the limited amount of primary sample material, the low number of acetylation sites within the FoxP3 protein, and the fact that acetylation only occurs in a small fraction of a protein population [65]. Thus, although we could not detect FoxP3-specific changes in acetylation/ubiquitination, we cannot exclude that this effect still contributes to the higher FoxP3 levels seen upon ACC1 inhibition.

Histone acetylation is highly sensitive to the abundance of acetyl-CoA and is closely linked to enhanced gene transcription by rendering the chromatin more accessible for transcription factors and polymerases [26,66]. Consistent with previous reports [23,25], we found that ACC1 inhibition promoted global histone acetylation in Tregs and sustained Foxp3 transcription. Specifically in the *Foxp3* locus, absence of ACC1 elevated the acetylation of histone H3K27, particularly in the TGF-β-responsive CNS1 region, which enables the formation of iTregs even in the absence of TSDR demethylation [[67], [68], [69]]. This epigenetic remodelling of the *Foxp3* locus was sufficient to promote stable FoxP3 expression in both iTregs and *ex vivo*-expanded nTregs, supporting the notion that ACC1 inhibition might also enhance Treg stability by driving sustained FoxP3 transcription through histone acetylation.

Furthermore, our data show that this Treg-promoting effect is mediated by acetyl-CoA-dependent mechanisms, which we confirmed by demonstrating that SorA's effects were partially dependent on two key enzymes: ATP citrate lyase (ACLY) [55], which generates acetyl-CoA from citrate after export from the mitochondria, and the HAT p300, which mediates both FoxP3 acetylation and histone acetylation at the *Foxp3* locus [48]. Inhibition of these enzymes abolished SorA-induced Treg differentiation, suggesting that ACC1 inhibition functions by increasing acetyl-CoA availability. These findings align with previous studies linking cytosolic acetyl-CoA levels to epigenetic regulation of T effector cells, including Th1, Th17, and cytotoxic CD8^+^ T cells [[70], [71], [72], [73], [74], [75], [76]].

Adoptive Treg therapy holds great promise for treating autoimmune and inflammatory diseases. *Ex vivo*-expanded nTregs have shown encouraging results in early human trials for graft-versus-host disease (GVHD), but the limited availability of donor nTregs and their potential inferiority to antigen-specific or (CAR)-engineered iTregs have hindered broader application [[77], [78], [79], [80], [81]]. iTregs, which can be generated in large numbers from naïve CD4^+^ T cells, offer an alternative, but their therapeutic use has been limited by their instability and loss of FoxP3 expression [[82], [83], [84]]. Our study provides compelling evidence that ACC1 inhibition during iTreg differentiation enhances their stability and FoxP3 retention, even after adoptive transfer in models of inflammatory diseases such as EAE and colitis. This suggests that modulating metabolic pathways can improve the efficacy of adoptive Treg therapies.

One attractive option would be combining SorA with other epigenetic modulators promoting acetylation, like retinoic acid [72] and HDAC inhibitors [85], or demethylation, like 5-azacytidine [30], to generate stable antigen-specific iTregs.

In conclusion, we identified ACC1 as a dual metabolic and epigenetic regulator during T-cell differentiation. By increasing acetyl-CoA availability, ACC1 inhibition enhances genome-wide chromatin accessibility and histone acetylation at the *Foxp3* locus, promoting sustained FoxP3 expression and long-term Treg stability. These findings position ACC1 as a promising metabolic target for improving adoptive Treg therapies in autoimmune diseases and graft rejection, offering new strategies to enhance the effectiveness of Treg-based immunotherapies.

## 4 Material and methods

### 4.1 Ethic statement

All animal experiments were performed in compliance with the German animal protection law (TierSchG BGBl. I S. 1105; 25.05.1998). The mice were housed and handled in accordance with good animal practice as defined by FELASA and the national animal welfare body GV-SOLAS. All animal experiments were approved by the Lower Saxony Committee on the Ethics of Animal Experiments as well as the responsible state office (Lower Saxony State Office of Consumer Protection and Food Safety) under the permit numbers 33.19-42502-04-15-1851 and 33.19-42502-04-18/2849 considering the German Animal Welfare Act.

### 4.2 Mice

C57BL/6 mice were purchased from Jackson Laboratories or bred in-house. TACC1 mice were generated by crossing ACC1^flox^ mice [41] to CD4-cre mice [86] and maintained on a C57BL/6 genetic background, as described previously [12]. ACC2^ko^ [87] were backcrossed to the C57BL/6 background and crossed to TACC1 mice generating TACC1 × ACC2^*ko*^ mice. DEREG [3], FoxP3^RFP^ [88], Thy1.1, and Rag2^ko^ mice were bred on the C57BL/6 background. Sex- and age-matched littermates between 8 and 16 weeks of age were used for all experiments. All mice were bred and kept under specific pathogen-free conditions at the animal facility of the Helmholtz Center for Infection Research (HZI, Braunschweig, Germany) or TWINCORE (Hannover, Germany). TACC1 and DEREG mice were also bred and housed in the animal facility of the University Medical Center of the Johannes Gutenberg. University of Mainz.

### 4.3 T cell cultures

CD4^+^ T cells were isolated *ex vivo* from spleens and lymph nodes of mice by enrichment with Dynal Mouse CD4 Negative Isolation Kit (Life Technologies) followed by FACS sorting (FACSAria Fusion or FACSAria, BD; XDP or MoFlo, Beckman Coulter) for live CD4^+^CD25^−^CD62L^+^ naïve T cells with >95% purity. IMDM GlutaMAX medium or RPMI 1640 GlutaMAX medium (both Thermo Fisher Scientific) was used for Th17 or Treg cultures, respectively. Medium was supplemented with 10% heat-inactivated FCS (Biochrom), 500 U penicillin-streptomycin (PAA Laboratories), and 50 μM β-mercaptoethanol (Life Technologies). For Th17 cell induction, 2–3 × 10^5^ naïve T cells were cultured for 4 days with plate-bound anti-CD3ε (10 μg/mL, clone 145-2C11; Bio X Cell) in the presence of soluble anti-CD28 (1 μg/mL, clone 37.51; Bio X Cell), anti–IFN–γ (5 μg/mL, clone XMG1.2; Bio X Cell), anti-IL-4 (5 μg/mL, clone 11B11; Bio X Cell), rhTGF-β1 (2 ng/mL; Peprotech), rmIL-6 (5 ng/mL; Peprotech), and rmIL-1β (50 ng/mL; Peprotech). For Treg cell induction, 2.5 × 10^4^ naïve T cells were cultured for 4 days with plate-bound anti-CD3ε (5 μg/mL) in presence of soluble anti-CD28 (1 μg/mL), rhIL-2 (200 U/mL; Roche Applied Science), and optimal (1–0.5 ng/mL) or suboptimal (0.25–0.33 ng/mL) concentrations of rhTGF-β1. rhIL-2 was added again on day 2. SorA (standard experiments: 200 nM, transfection experiments: 1 μM; Helmholtz Zentrum, Saarbrücken) and [U–^13^C_6_] glucose (1 mM; Cambridge Isotope Laboratories) were added at the onset of the cultures. To block acetylation, chemical inhibitors of the HAT p300 (C646; Sigma–Aldrich) or ACLY (BMS 303141) were added at the start of the culture. When indicated, naïve T cells and nTregs were labelled using 5 μM CellTrace Violet Cell Proliferation Kit (Life Technologies) in order to assess their proliferation.

### 4.4 Treg stability

iTregs were generated from DEREG mice in the presence or absence of SorA as described above, under optimal (1 ng/mL) or suboptimal (0.25–0.33 ng/mL) rhTGF-β1 concentrations. On day 4, GFP^+^FoxP3^+^ iTregs were re-sorted and re-plated in the presence of rhIL-2 (200 U/ml) with or without rhTGF-β (1 ng/mL) in U-bottom plates coated with anti-CD3ε (1 μg/mL) and anti-CD28 (1 μg/mL). After 3 days, cells were removed from stimulation by transferring them to new uncoated U-bottom plates. Every two days, rhIL-2 (200 U/ml) was added. At indicated time points, FoxP3 expression was determined by flow cytometry. nTregs were isolated by sorting CD4^+^CD25^hi^ T cells from WT mice or CD4^+^GFP^+^ T cells from DEREG mice by FACS sorting. Cells were expanded *ex vivo* with plate-bound anti-CD3ε (1 μg/mL, clone 17A2; eBioscience/Thermo Fisher Scientific) and anti-CD28 (1 μg/mL, clone 37.51; eBioscience/Thermo Fisher Scientific) supplemented with rhIL-2 (400 U/ml) in the presence of DMSO or SorA in F-bottom plates (non-treated surface; Thermo Fisher Scientific). On day 4, cells were removed from stimulation by transferring them to new F-bottom plates (non-treated surface). Every two days, rhIL-2 (400 U/ml) was added. At indicated time points, cells were harvested and lysed for RNA isolation or stained for flow cytometric analysis of FoxP3 expression. To evaluate the effects of ubiquitination on Treg stability, nTregs were harvested on day 4 of *ex vivo* expansion, counted and cultured with an inhibitor (DUBI) of the deubiquitinase USP7 (P5091; Selleckchem). After 5 h, cells were lysed, and whole-cell lysates were subjected to immunoblotting for FoxP3 and ubiquitin.

### 4.5 Flow cytometry

The following monoclonal antibodies specific to mouse antigens and labelled with the indicated fluorescent markers were purchased from eBioscience/Thermo Fisher Scientific: CD4 eFluor450 (RM4-5), CD4 Alexa488, CD4 eFluor660 (both GK1.5), FoxP3 eFluor450, FoxP3 eF660 (both FJK-16s), CD62L PE-Cy7 (MEL-14), IL-17A APC, IL-17A PE-Cy7 (both eBio17B7), IFN-γ PE (XMG1.2), RORγt APC and PE (B2D), CD25 PE, CD25 APC (both PC61.5), CD45RB PE (C363.16A), Thy1.1 APC (HIS51), Thy1.2 PE-Cy7 (53–2.1). To analyse intracellular cytokine production, T cells were stimulated with phorbol 12-myristate 13-acetate (PMA, 100 ng/mL; Sigma–Aldrich) and ionomycin (1 μg/mL; Sigma–Aldrich) for 2 h, followed by an additional 2 h in the presence of Brefeldin A (5 μg/mL; eBioscience/Thermo Fisher Scientific). Intracellular staining for transcription factors, acetylated-Lysine (Cell Signaling Technology) and cytokines was performed by using the FoxP3/Transcription Factor Fixation/Permeabilisation Kit (eBioscience/Thermo Fisher Scientific) according to the manufacturer's instruction. Lipid uptake was determined by incubating cells in 100 μL PBS with 1 μg/mL Bodipy FL C_16_ (Thermo Fisher Scientific) at 37 °C for 30 min. The accumulation of lipids was evaluated using HCS LipidTOX™ Phospholipidosis and Steatosis Detection Kit (Thermo Fisher Scientific), which contains LipidTOX™ Red phospholipid stain and LipidTOX™ Green neutral lipid stain. For the detection of phospholipids, LipidTOX™ Red was added at the start of the culture. For assessing the accumulation of neutral lipids, cells were stained with LipidTOX™ Green neutral lipid stain after harvesting and fixation with paraformaldehyde (2%; Carl Roth) according to the manufacturer's instructions. Data acquisition was conducted on a CyAn ADP (Beckman Coulter), LSR II (Becton Dickinson) or CytoFLEX S (Beckman Coulter), and data were analysed with FlowJo software (Becton Dickinson).

### 4.6 Transfection and immunoprecipitation

293T cells were cultured in DMEM (Thermo Fisher Scientific) containing 10% heat-inactivated FCS (Biochrom), 500 U penicillin-streptomycin (PAA Laboratories) and 10 mM HEPES (Thermo Fisher Scientific). For transfection, 293T cells were seeded in 6-well plates overnight to achieve 70–80% confluency. The next day, 293T cells were transfected with expression vectors for FLAG-FoxP3 (kindly provided by Dr. Melanie Ott) and myc-p300 (kindly provided by Dr. Eric Verdin) in 1:4 ratio by using calcium phosphate precipitation in the presence of 25 mM chloroquine (Sigma). H_2_O served as a negative transfection control. 6–8 h after transfection, the culture medium was replaced, and cells were cultured for another 18 h in the presence or absence of SorA (1 μM; Helmholtz Zentrum, Saarbrücken). Cells were harvested in p300 lysis buffer 250 mM NaCl (Carl Roth), 1% Triton-X100 (Sigma–Aldrich), 7.6 mM NaH_2_PO_4_ (sodium dihydrogen phosphate), 12.4 mM Na_2_HPO_4_ (disodium hydrogen phosphate; both Carl Roth), 30 mM Na_2_H_2_P_2_O_7_ (sodium pyrophosphate dibasic; Sigma–Aldrich), 5 mM EDTA (Carl Roth) supplemented with 400 nM TSA, 5 mM Nicotinamide (both Sigma–Aldrich), 10 mM NaF (Merck Millipore), and complete EASYpack Mini Protease Inhibitor Cocktail (Roche Applied Science). Lysates were immunoprecipitated with anti-FLAG® M2 affinity gel (Sigma–Aldrich), washed with lysis buffer and eluted with FLAG peptide (150 μg/mL). Samples were boiled in Laemmli sample buffer for SDS-PAGE and subjected to immunoblotting.

### 4.7 Western blot

Cytosolic and nuclear cell lysates were generated using a combination of made-in-house isotonic lysis buffer (250 mM Sucrose, 10 mM HEPES, 2 mM MgCl_2_) and nuclear extraction buffer (20 mM Tris pH = 8, 100 mM NaCl, 2 mM EDTA pH = 8). The lysates for each fraction were prepared through a series of steps, including the snap-freezing of samples on liquid nitrogen and subsequent heating at 37 °C. In all cases, the lysis buffer for each fraction was supplemented with complete EASYpack Mini Protease Inhibitor Cocktail and PhosSTOP Phosphatase Inhibitor (both Roche Applied Science). Whole-cell lysates were prepared using lysis buffer (Pierce RIPA buffer, Thermo Scientific) supplemented with the cocktail of inhibitors mentioned above. Cell lysates were separated by SDS-gel electrophoresis and transferred to polyvinylidene fluoride membranes (Merck Millipore). Immunoblotting was performed using GAPDH (clone D16H11; 1:1000) (Cell Signalling Technology), FoxP3 (clone eBio7979; 1:750) (eBioscience/Thermo Fisher Scientific), β-actin (clone AC-15; 1:20.000), anti-FLAG M2 (clone M2; 1:1000) (both Sigma–Aldrich), mono- and polyubiquitinylated conjugates monoclonal antibody (clone FK2; 1:1000; Enzo Life Science), goat anti-rabbit horseradish peroxidase, and goat anti-mouse horseradish peroxidase (both Jackson ImmunoResearch). The anti-AcK31 FoxP3 antibody was kindly provided by Dr. Melanie Ott (self-made). Western Blots were detected using Pierce™ ECL Western Blotting Substrate, SuperSignal™ West Pico Chemiluminescent Substrate, or SuperSignal™ West Femto Maximum Sensitivity Substrate (all Thermo Fisher Scientific) with the ChemoStar system (Intas).

### 4.8 Ubiquitin-mediated degradation of FoxP3

To evaluate the effects of ubiquitination on Treg stability, *ex vivo-*expanded nTreg or iTregs were harvested on day 4, counted, and cultured with 10 μM of P5091, an inhibitor of the deubiquitinase USP7 (DUBI; Selleckchem). After 5 h, cells were lysed, and whole-cell lysates were subjected to immunoblotting for ubiquitin and FoxP3.

### 4.9 Gene expression analysis

Cells were lysed in TRIzol reagent (Thermo Fisher Scientific), and RNA was isolated with Direct-zol RNA MiniPrep (Zymo Research). 1 μg of total RNA was retro-transcribed into cDNA using SuperScript III Reverse Transcriptase Kit (Thermo Fisher Scientific). Real-time PCR reactions were carried out using Fast SYBR Green Master Mix (Bio-Rad) in a Light Cycler 480 [89]. Gene expression was normalised to the housekeeping gene *Actb*.

### 4.10 ^13^C incorporation assays

For ^13^C incorporation analysis, [U–^13^C_6_] glucose (1 mM; Cambridge Isotope Laboratories) was added at the onset of *in vitro* T cell cultures. To determine the incorporation of glucose-derived carbon into cellular FAs, cells were saponified (MeOH:NaOH (15%) 1:1, 1 h, 100 °C), derivatised (MeOH:HCl 10:2, 10 min, 80 °C) and then prepared for analysis using a gas chromatography-combustion-isotope ratio mass spectrometer (GC/C/IRMS) as described earlier [90]. GC/C/IRMS measurements were performed in triplicate on a Finnigan MAT 253 isotope ratio mass spectrometer coupled with a Trace GC Ultra (Thermo Fisher Scientific) chromatograph via a combustion interface. The FA methyl esters were separated with an Optima five column (5% phenyl, 95% dimethylpolysiloxane, 50 m, 0.32 mm inner diameter, and 0.25 μm film thickness). The oven program was 100 °C for 2 min, increased to 290 °C at 4 °C min^−1^, followed by an isothermal period of 8 min. The separated compounds were combusted on line in an oxidation oven. ^13^C/^12^C isotope ratios for the free FAs were calculated as described [90] and are presented as δ^13^C incorporation in the figures.

### 4.11 Acetyl-CoA quantification by high-performance liquid chromatography-MS (HPLC-MS/MS)

Cells were harvested after 24 h of culture and resuspended in a solution of acetonitrile, methanol, and water (2:2:1, v/v; all HPLC grade), supplemented with the internal standard 13C3-malonyl-CoA. The combined extracts, obtained after repeated extraction, were stored overnight at −20 °C to allow complete protein precipitation. After thawing, samples were centrifuged at 20,800×*g* for 10 min at 4 °C. The supernatants were transferred to Eppendorf tubes and dried using a vacuum concentrator (Centrivap, LabConco). Simultaneously, the protein concentration was determined from the pellets after drying and resuspension in 0.1 M NaOH, using a bicinchoninic acid assay kit (Thermo Scientific) according to the manufacturer's instructions. Protein concentrations were used to normalize the relative concentrations of metabolites, expressed as peak area counts per microgram of protein.

The dried metabolite extracts were reconstituted in 50 μL of the mobile phase A and transferred to LC vials. A 10 μL volume was injected into an Agilent 1260 Infinity II UPLC system (Agilent Technologies, CA, USA) equipped with a Waters Acquity UPLC HSS T3 column (1.8 μm, 2.1 × 150 mm) maintained at 40 °C. The mobile phase consisted of Buffer A (5 mM ammonium acetate in water, pH 6.8) and Buffer B (100% methanol). The 40-minute gradient program, with a flow rate of 0.3 mL/min, started at 2% Buffer B, ramped up to 98% over 20 min, was held for 5 min, and then returned to 2% Buffer B for equilibration. Mass detection of acetyl-CoA was performed using a QTRAP5500 triple quadrupole mass spectrometer (AB Sciex) equipped with an electrospray ionization (ESI) source operating in positive ionization mode. Multiple reaction monitoring (MRM) analysis was applied with the following mass transitions: acetyl-CoA, *m*/*z* 810.10 → 303.10, and 13C3-malonyl-CoA (internal standard), *m*/*z* 857.2 → 350.30. Mass spectral data were processed using Analyst software (Sciex) to obtain peak areas of the respective compounds in .csv format.

### 4.12 Chromatin immunoprecipitation (ChIP)

For ChIP analysis of histone acetylation in the *Foxp3* locus, nTregs and iTregs were expanded or generated as described above and harvested at the indicated time points. ChIP was performed with the MAGnify Chromatin Immunoprecipitation System (Thermo Fisher Scientific). Briefly, 0.5 × 10^6^ cells were fixed with 1% paraformaldehyde at room temperature for 10 min. After washing with ice-cold PBS, the cells were lysed in lysis buffer (50 mM Tris-Cl pH 8, 10 mM EDTA, 1% SDS). Chromatin was sheared into ∼400 bp fragments by using a Bioruptur® sonicator. To prevent unspecific binding, sheared chromatin (3.75 μg) was pre-cleared with beads for 1 h at 4 °C, and beads were removed before immunoprecipitation. Anti-acetylated H3K27 (H3K27ac) (1.2 μg, ab4729; Abcam) or rabbit IgG (1.2 μg, MAGnify Chromatin Immunoprecipitation System; Thermo Fisher Scientific) antibodies were conjugated with beads for 4 h at 4 °C. For immunoprecipitation, pre-cleared chromatin was incubated overnight at 4 °C with antibody-bead conjugates. After reverse crosslinking, DNA was purified with the QIAquick PCR purification kit (Qiagen) and was analysed by RT-PCR using the following primers (Eurofins Genomics):

*Foxp3* Promoter-forward: CTGAGGTTTGGAGCAGAAGGA

*Foxp3* Promoter-reverse: TCTGAAGCCTGCCATGTGAA

*Foxp3* CNS1-forward: ACTTAGTTTATGAGCATGCATGTTCTTC

*Foxp3* CNS1-reverse: TGAGATCCCACACCATCTTCTG

*Foxp3* CNS2-forward: GTTGCCGATGAAGCCCAAT

*Foxp3* CNS2-reverse: ATCTGGGCCCTGTTGTCACA

### 4.13 Bulk ATAC and ChIP sequencing

iTregs treated with vehicle or SorA from WT or TACC1 mice were harvested after two days. For each ATAC reaction 5 × 10^4^ cells were sorted into a tube and spun down at 500×*g* for 5 min at 4 °C, before the supernatant was discarded completely. Cells were washed with PBS and lysed by resuspension in a lysis buffer with 0.1% NP-40 and 0.01% Digitonin for 3 min on ice. Lysis was stopped by adding ATAC-Resuspension Buffer (10 mM TrisHCl, 10 mM NaCl, 0.1% Tween-20) and centrifugation at 500*g* for 10 min at 4 °C. Tagmentation was performed in 1× Tagmentation Buffer (Illumina) with 0.01% Digitonin using Tagment DNA TDE1 Enzyme (Illumina) at 37 °C for 30 min while shaking at 1000 rpm. DNA was purified using Monarch PCR & DNA clean-up columns (NEB) and libraries were prepared by PCR (11 cycles) using Phusion polymerase (NEB) and IDT for llumina UD Indexes (Illumina) with 1.18 M Betaine. Libraries were cleaned up using Ampure XP beads, initially at 1.8× ratio, followed by a right sided 0.55× ratio size selection and a second 1.8× ratio clean-up. Libraries were quantified using Qubit HS dsDNA and TapeStation HSD1000 reagents and paired-end sequenced on an Illumina NextSeq2000. Chromatin immunoprecipitation (ChIP) was performed in triplicates as described previously with slight modifications [91]. Briefly, 2 × 10^6^ iTreg cells were mixed with 1 × 10^5^ HEK293T human cells (spike-in control) and crosslinked with 1% formaldehyde for 10 min at room temperature and the reaction was quenched with glycine at a final concentration of 0.125 M. Chromatin was sheared using the Covaris S220 focused-ultrasonicator to an average size of 250–350 bp. ChIPs were conducted using 20 μL magnetic Protein-A DynaBeads (Thermo Fisher Scientific) coupled to 2.5 μg of antibody. Samples were incubated for 3 h while rotating at room temperature. Beads were washed and crosslinks were reversed at 65 °C with 0.25 M NaCl overnight. After RNase A and proteinase K treatment, DNA was extracted with the Monarch PCR & DNA Cleanup kit (NEB). Sequencing libraries were prepared with the NEBNext Ultra II DNA Library Prep Kit for Illumina (NEB) according to the manufacturer's instructions. The quality of dsDNA libraries was analysed using the High Sensitivity D1000 ScreenTape Kit (Agilent) and concentrations were assessed with the Qubit dsDNA HS Kit (Thermo Fisher Scientific). Libraries were sequenced on a NextSeq2000 (Illumina).

### 4.14 ATAC and ChIP seq data processing

Analysis of ATAC-seq data was performed as described [92]. Briefly, paired-end reads were aligned to the mouse genome (GRCm38/mm10) using bowtie2, keeping only unique reads. Read positions were adjusted to move the ends proximal to the Tn5binding site. Initial QC and peak calling were performed as described earlier [92]. Statistically significant differences in read counts across peaks between sets of replicate ATAC-seq experiments were determined with quantile (0.95) regression and GC correction using edgeR (v3.42.1) with the cqn package in R (4.3.0).

ChIP-seq data analysis was performed as described [92] with modifications. Single-end reads were aligned to the mouse genome (GRCm38/mm10) and to the human genome (GRCh38.p10) using bowtie2, keeping only unique reads. Peaks were called separately across mouse samples and across merged human spike-in samples using HOMER's findPeaks program (v5.1) and parameters “-region -size 250 -L 0 –F 5 -minDist 350 -fdr 0.0001” and “-ntagThreshold 5” for human spike-in samples and “-ntagThreshold 10” for mouse samples. Peak sets were filtered by subtracting blacklisted genomic regions, and by filtering out regions with a mappability <0.8. Comparisons were performed in edgeR after calibrating the read counts for individual samples using the corresponding human spike-in read counts. The principle components were calculated in R from the top 500 variable peaks using the prcomp function in the stats package and plotted using ggplot2 (v3.4.2).

Read coverage across individual peaks sets were calculated using HOMER's annotatePeaks.pl with parameters “-hist 25 -ghist” using normalized replicate data sets. For histograms average read coverage data and 95% confidence intervals were calculated in R and the ggplot2 package was used to draw histograms. MvA plot were generated using ggplot2 in R. Genome tracks of ChIPseq and ATACseq data were generated using the pyGenomeTracks software (v3.5).

### 4.15 T cell transfer colitis

Similarly to previously published protocols [93,94], naïve CD4^+^CD25^−^CD45RB^hi^CD90.1^+^ T cells (0.4 × 10^6^ cells) from Thy1.1 mice were transferred i.p. alone or together with re-sorted FoxP3^+^GFP^+^ iTregs (0.16 × 10^6^ cells) in a ratio of 2.5:1 into RAG2^−/−^ mice. FoxP3^+^GFP^+^ iTregs were generated in the presence of DMSO or SorA. The weight of the mice was monitored twice a week, and after six to seven weeks, mice were euthanized and tissues harvested for histopathological and immunological analyses.

### 4.16 Histopathological scoring

For histological analysis, colon tissue was fixed in paraformaldehyde. Samples were further processed to paraffin blocks, sectioned, and stained with hematoxylin and eosin (H&E) at the Mouse Pathology Department of the MHH. Samples were examined in a blinded manner and scored as described previously [11]. In brief, tissue damage and cell infiltration were assessed and scored. Both scores were added, and the combined histological score ranged from 0 (no changes) to 6 (extensive cell infiltration and tissue damage).

### 4.17 Cell isolation from colon and small intestine

After washing with PBS and removing feces, colons were cut into several pieces and incubated in 20 mL of PBS/5 mM EDTA for 30 min at 37 °C on a shaker. Next, tissues were detached from the mucus, rinsed with ice-cold PBS, and mechanically dissociated, followed by enzymatic digestion in DMEM supplemented with 2% FCS, 1 mg/mL collagenase D [89] and 0.1 mg/mL DNase I [89] for 30 min at 37 °C. Tissue suspensions were passed through 100-μm strainer, pelleted, resuspended in 40% Percoll (GE Healthcare), and underlain with 80% Percoll. After centrifugation at 900*g* for 25 min at room temperature, cells were yielded from the interface of 40–80% Percoll gradient. Cells were washed with complete medium and used for restimulation or direct analysis.

### 4.18 Experimental autoimmune encephalomyelitis (EAE)

EAE was induced by subcutaneous immunisation with 200 μg MOG_35–55_ peptide (Department of Chemical Biology, HZI Braunschweig) emulsified in complete Freund's adjuvant (Sigma–Aldrich) and intravenous injection of 200 ng pertussis toxin (Sigma–Aldrich) on day 0 and day 2. Disease severity was assessed by daily scoring in a blinded manner with the following scale: 0, no paralysis; 0.5, clumsy gait; 1, limp tail; 2, limp tail and partial hind leg paralysis; 3, complete hind leg paralysis; 4, tetraparesis; 5, moribund. Animals were euthanized if scores reached grade 3.5 or remained at 3 more than two days and subsequently scored as 5 the following days. To assess the therapeutic effect of Treg transfer, 1 × 10^6^ iTregs, generated in the presence of DMSO or SorA and re-sorted for GFP^+^FoxP3^+^ cells, were adoptively transferred i.v. on day 3.

### 4.19 Statistical analysis

Data analyses were performed using GraphPad Prism Software version 7 (GraphPad Software), and statistics were performed as indicated in the figure legends. Statistical analyses were performed as follows: two-way ANOVA followed by Bonferroni's multiple comparison was used to analyse experiments with two variables and three or more groups; one-way ANOVA followed by Bonferroni's multiple comparison was used for experiments with one variable and three or more groups; unpaired Student's t-test and paired t-test were used to compare two groups as indicated in the figure legends. Means are given as ±s.d. or, where indicated, as ±s.e.m., with *P* values considered significant as follows: ∗*P* < 0.05; ∗∗*P* < 0.01, ∗∗∗*P* < 0.001 and ∗∗∗∗*P* < 0.0001 or n.s. (not significant).

## Funding

This work was supported by the joined grant (SP615/12-1) of the Deutsche Forschungsgemeinschaft (DFG) and the French Agence Nationale de la Recherche (ANR) to LB, TS, and BLS as well as a grant from the Deutsche Forschungsgemeinschaft Project-ID 490846870 - TRR355 TPB08 and IRTG to LB, CRC156, Project-ID 318346496 – SFB 1292 TP18 and SFB TRR355 TPA04 to TS. LB was funded by the Ellen-Schmidt Program and Hochschulinterne Förderung (HiLF) from the Hannover Medical School. PS was supported by the International Research Training Group 1273 from the DFG and the DFG/ANR grant (SP615/12-1). Florencia Hellriegel was supported with a Travel Grant from the Boehringer Ingelheim Foundation.

## CRediT authorship contribution statement

**Philipp Stüve:** Formal analysis, Investigation, Methodology, Visualization, Writing – original draft, Writing – review & editing. **Gloria J. Godoy:** Formal analysis, Investigation, Methodology, Visualization, Writing – original draft, Writing – review & editing. **Fernando N. Ferreyra:** Investigation. **Florencia Hellriegel:** Formal analysis, Investigation. **Fatima Boukhallouk:** Investigation. **Yu-San Kao:** Investigation. **Tushar H. More:** Investigation, Methodology. **Anne-Marie Matthies:** Investigation. **Tatiana Akimova:** Investigation. **Wolf-Rainer Abraham:** Investigation, Resources. **Volkhard Kaever:** Investigation, Resources. **Ingo Schmitz:** Investigation, Resources. **Karsten Hiller:** Investigation, Resources. **Matthias Lochner:** Investigation. **Benoît L. Salomon:** Writing – review & editing. **Ulf H. Beier:** Investigation, Writing – review & editing. **Michael Rehli:** Data curation, Formal analysis, Methodology, Resources, Software, Visualization, Writing – review & editing. **Tim Sparwasser:** Conceptualization, Funding acquisition, Project administration, Resources, Supervision. **Luciana Berod:** Conceptualization, Funding acquisition, Project administration, Resources, Supervision, Visualization, Writing – review & editing.

## Declaration of competing interest

The authors declare the following financial interests/personal relationships which may be considered as potential competing interests: Luciana Berod reports financial support was provided by German Research Foundation. If there are other authors, they declare that they have no known competing financial interests or personal relationships that could have appeared to influence the work reported in this paper.

## Data Availability

Data will be made available on request.
