# Supplementary material for: ACC1 is a dual metabolic-epigenetic regulator of Treg stability and immune tolerance
Source: Mol Metab. 2025 Feb 8;94:102111. doi: 10.1016/j.molmet.2025.102111 (PMC11893314; doi:10.1016/j.molmet.2025.102111)
Supplement: Multimedia component 1 [file mmc1.docx]

**Supplemental information**

**Supplementary Figures**

**
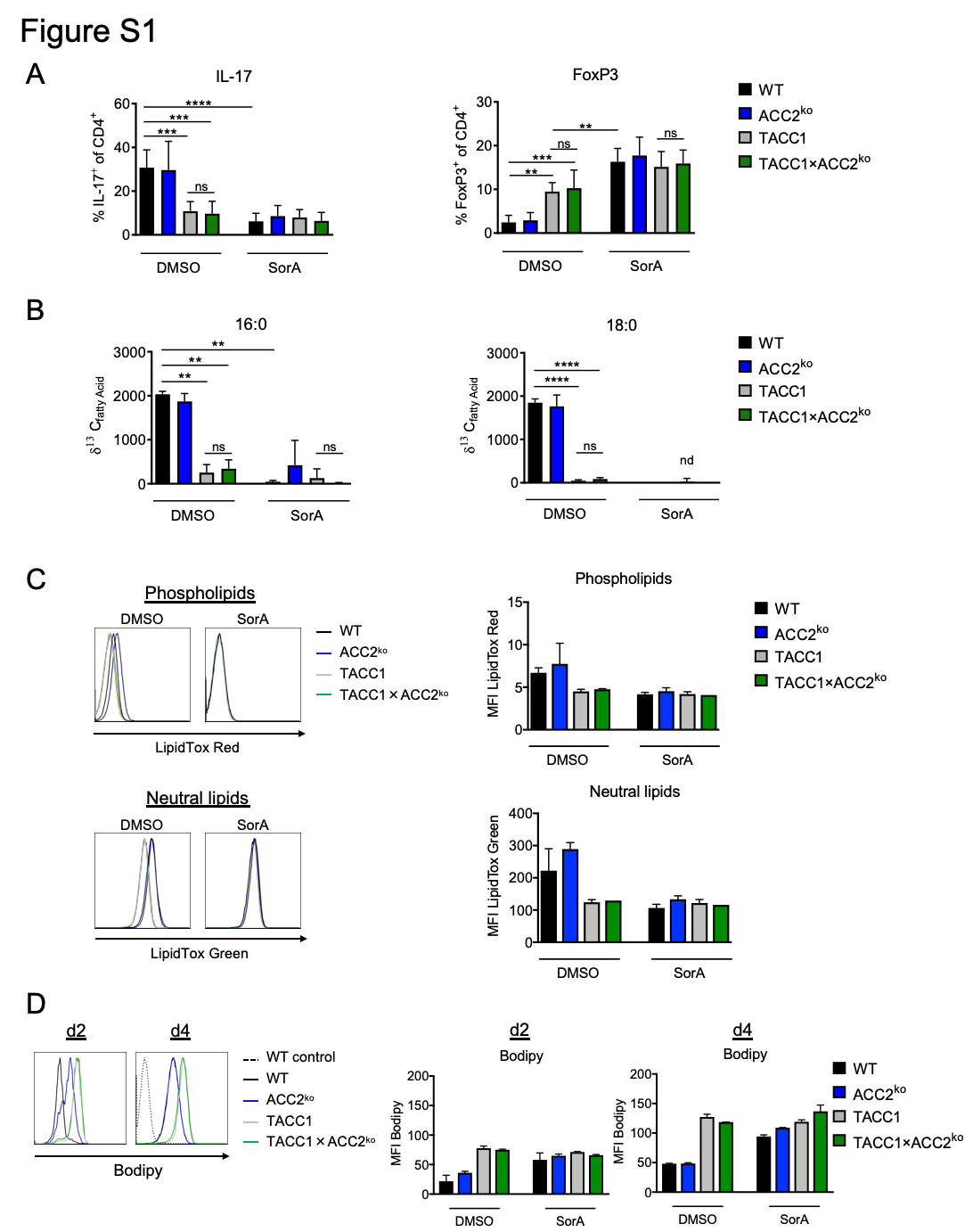
**

**Figure S1: Inhibition of ACC1, but not ACC2, promotes Treg development.** (A-D) Naïve CD4^+^ T cells from WT, ACC2^ko^, TACC1, and TACC1 × ACC2^ko^ mice were cultured under Th17-polarizing conditions in the presence of DMSO or SorA and analysed by flow cytometry (A, C, D) or mass spectrometry (B). (A) Frequency of IL-17^+^ and FoxP3^+^ cells among live CD4^+^ T cells on day 4 of culture. (B) Incorporation (δ) of ^13^C into FAs in the presence of [U-^13^C_6_] glucose on day 4 of culture. n.d., not detected. (C) Accumulation of phospholipids (left) and neutral lipids (right) assessed by LipidTox Red and LipidTox Green staining after 62 h of culture. Bar graphs show the mean of fluorescence (MFI) of LipidTox Red/Green. (D) Bodipy FL C_16_ uptake was determined on day 2 and day 4 of culture. Bar graphs show the MFI of Bodipy^+^ cells among live CD4^+^ T cells. Results are from one (C: phospholipids SorA treatment) experiment or representative of two (C: neutral lipids, D) independent experiments or shown as pooled data from four to seven (A) or three (B) independent experiments. Error bars represent s.d. of duplicates (C, D) and pooled data (A, B). **P < 0.01, ***P < 0.001, ****P < 0.0001. n.s., non-significant. One-way ANOVA with Bonferroni correction (A, B, C)

**
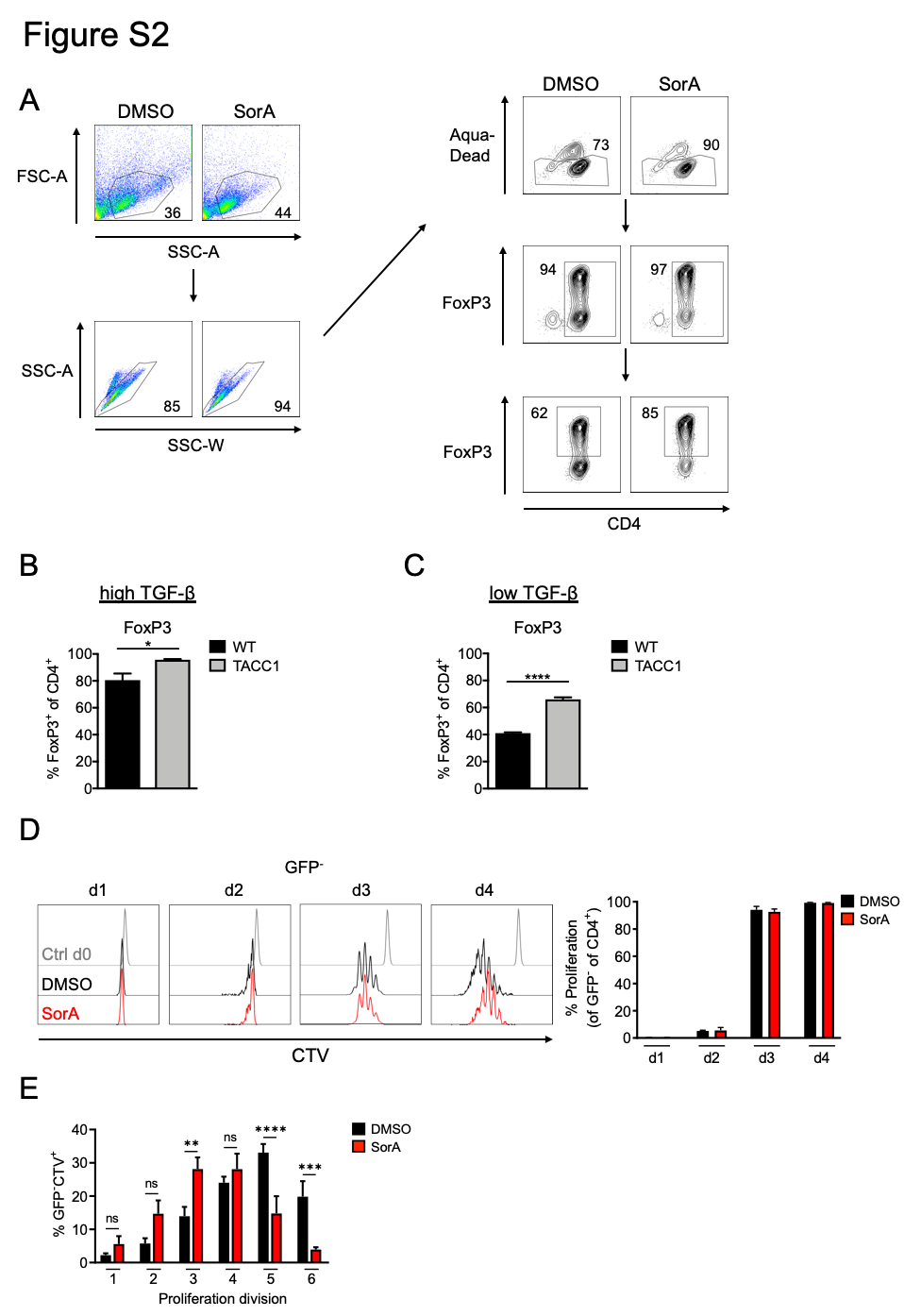
**

**Figure S2: Absence of ACC1 activity promotes Treg differentiation in iTreg cultures.** (A) Representative gating strategy for the analysis of iTregs generated in the presence of DMSO or SorA on day 4 of culture by flow cytometry. (B, C) Naïve CD4^+^ T cells from WT or TACC1 mice were cultured under iTreg-polarizing conditions using high (B) or low (C) TGF-β concentrations. Bar graphs display the frequency of FoxP3^+^ cells among live CD4^+^ T cells on day 4 of culture determined by flow cytometry. (D, E) Naïve CD4^+^ T cells from DEREG mice were cultured under suboptimal iTreg-inducing conditions in the presence of DMSO or SorA and their proliferation was assessed by the cell proliferation dye CellTrace violet (CTV) over the course of the culture. (D) Bar graphs represent percentage of GFP^-^-proliferating cells at specified time points determined by flow cytometry. (E) Bar graphs show percentage of CTV^+^FoxP3-GFP^-^ cells among total live CD4^+^ T cells in each proliferation cycle. Results are representative of three (D, E), five (B, C) or six (A) independent experiments and error bars show the s.d. of triplicates (B, C) or pooled data (D, E). *P < 0.05, **P < 0.01, ***P < 0.001, ****P < 0.0001. n.s., non-significant. Two-tailed Student’s t-test (B, C) and two-way ANOVA with Bonferroni correction (D, E).

**
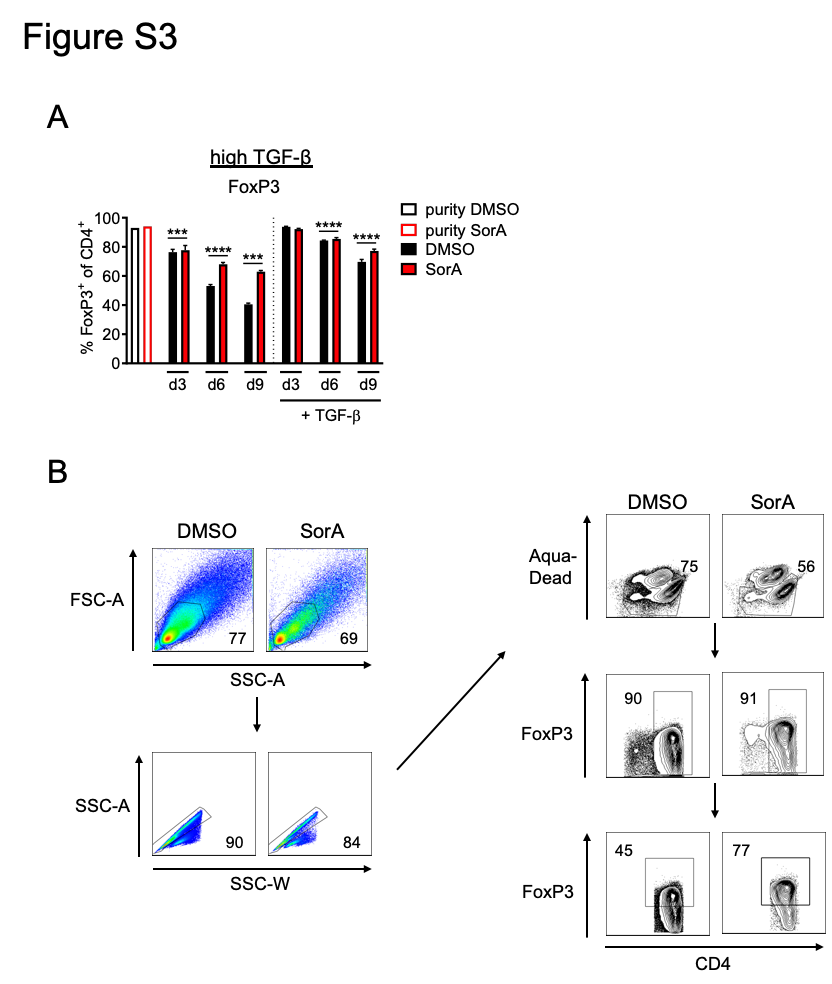
**

**Figure S3: Absence of ACC1 activity promotes Treg stability.** (A) Naïve CD4^+^ T cells from DEREG mice were cultured under iTreg-inducing conditions using a high TGF-β concentration in the presence of DMSO or SorA. After differentiation, GFP^+^FoxP3^+^ iTregs were re-sorted and plated in 200 U/ml IL-2 with or without TGF-β (1 ng/ml). FoxP3 levels in live CD4^+^ T cells were determined directly after re-sort (purity) and at different time points after re-plating by flow cytometry. (B) Representative gating strategy for the analysis of CD4^+^GFP^+^FoxP3^+^ nTregs isolated from DEREG mice and expanded *ex vivo* in the presence of DMSO or SorA on day 10 of culture by flow cytometry. Results are representative of three (A) or eight (B) independent experiments and error bars show the s.d. of triplicates (A). ***P < 0.001, ****P < 0.0001. Two-tailed Student’s t-test (A).


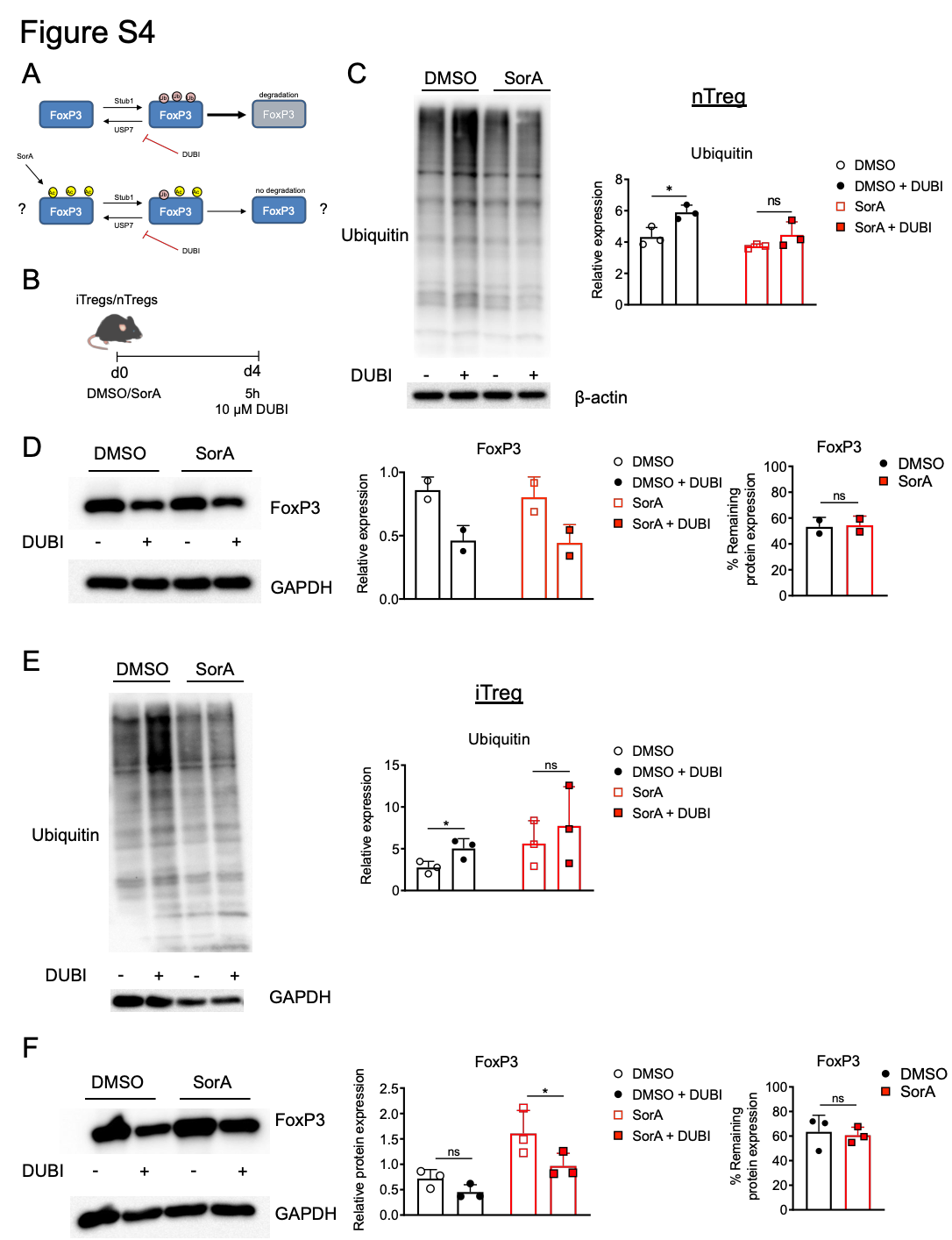


**Figure S4: Inhibition of ACC1 does not prevent ubiquitin-mediated degradation of FoxP3.** (A) Schematic depiction of the ubiquitin ligase Stub1 and the deubiquitinase USP7 reciprocally regulating FoxP3 protein stability by ubiquitin-mediated degradation, and a potential role of SorA. (B) Schematic depiction of the experimental layout. CD4^+^CD25^+^ nTregs were expanded *ex vivo* (C, D) and naïve CD4^+^ T cells were cultured under iTreg-inducing conditions using a low TGF-β concentration (E, F), both in the presence of DMSO or SorA. After 4 days, nTregs and iTregs were incubated in the presence or absence of the USP7 inhibitor P5091 (DUBI; 10 µM) for 5h. (C, E) Representative western blot depicts ubiquitinated proteins in whole-cell lysates of nTregs (C) and iTregs (E). Bar graphs show relative ubiquitination normalised to β-Actin. (D, F) Representative western blot depicts FoxP3 protein levels in nTregs (D) and iTregs (F). Bar graphs display the relative expression of FoxP3 protein normalised to GAPDH (left) and the frequency of remaining FoxP3 protein levels comparing DUBI-treated to untreated samples after normalisation to GAPDH loading control (right). Results are presented as pooled data from two (D) or three (C, E, F) independent experiments. Each symbol represents an individual experiment. Error bars indicate the s.d. of pooled data. *P < 0.05. n.s., non-significant. Two-tailed Student’s t-test (right: D, F) or Two-way ANOVA with Bonferroni correction (C and left: D,F).

**
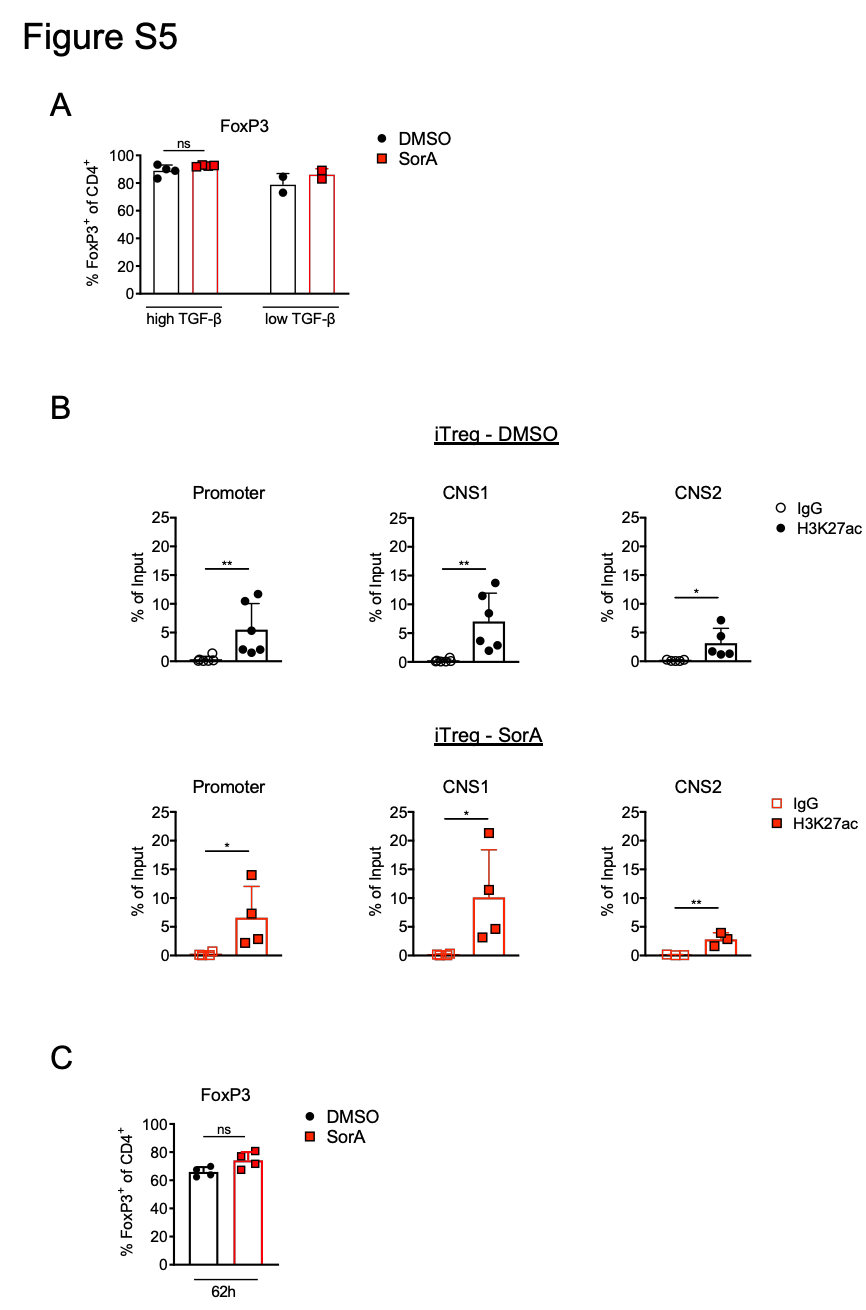
**

**Figure S5: ACC1 inhibition increases chromatin accessibility within the *Foxp3* locus.** (A) Naïve CD4^+^ T cells from DEREG mice were cultured under iTreg-inducing conditions using high or low TGF-β concentrations in the presence of DMSO or SorA. On day 4, GFP^+^FoxP3^+^ iTregs were re-sorted and FoxP3 expression was determined by FACS. (B, C) Naïve CD4^+^ T cells were cultured under iTreg-inducing conditions using a low TGF-β concentration in the presence of DMSO or SorA. (B) Cells were harvested after 62 h of culture and subjected to ChIP. Graphs show ChIP analysis of H3K27ac at the *Foxp3* locus. IgG serves as a negative control. Values are presented as % of input based on normalisation to input DNA. (C) The frequency of FoxP3^+^ cells was determined by flow cytometry after 62 h of iTreg culture. Results are pooled data from two to four (A), four to six (B, C) independent experiments. Each symbol represents an independent experiment. Error bars indicate s.d. of pooled data. *P < 0.05 **P < 0.01. n.s., non-significant. One-way ANOVA with Bonferroni correction (A), Two-tailed Student’s t-test (C: DMSO versus SorA) or One-tailed Student’s t-test (B: IgG versus H3K27ac).


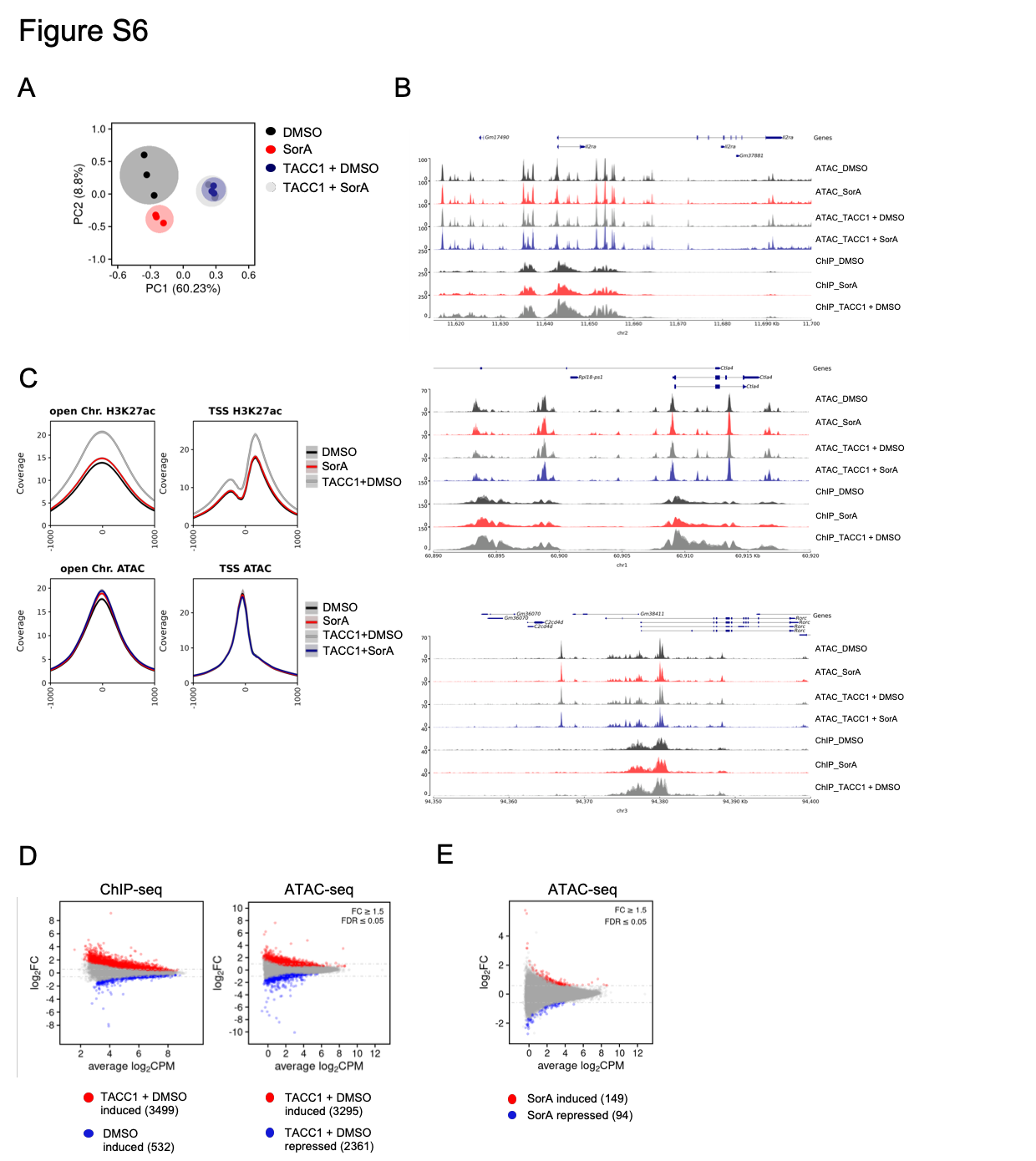


**Figure S6: Absence of ACC1 activity promotes chromatin accessibility and increased acetylation at H3K27 in iTreg cultures.** (A-E) Naïve CD4^+^ T cells from DEREG or TACC1 mice were cultured for 62 hours under iTreg-inducing conditions using a low TGF-β concentration in the presence of vehicle (DMSO) or SorA. Cells were analysed using ATAC-seq and histone H3K27 acetylation (ac) ChIP-seq analysis. (A) PCA of ATAC-seq using the top 500 variants regions. (B) H3K27ac ChIP-seq tracks and ATAC-seq derived accessibility tracks for *Il2ra, Ctla4* and *Rorc* loci. (C) Histograms plots showing calibrated H3K27ac signals across open chromatin regions overlapping with H3K27ac peaks, or across all transcription start sites (TSS) from DEREG- or TACC1-iTregs with and without SorA treatment. (D) MvA plots showing induced H3K27ac peaks (left) or differential accessible peaks (right) in vehicle treated-iTregs derived from DEREG and TACC1 mice. (E) MvA plot showing differential accessible peaks in vehicle or SorA-treated iTregs from DEREG mice. Data represent average coverage of three biological replicates per condition (n=3).

**
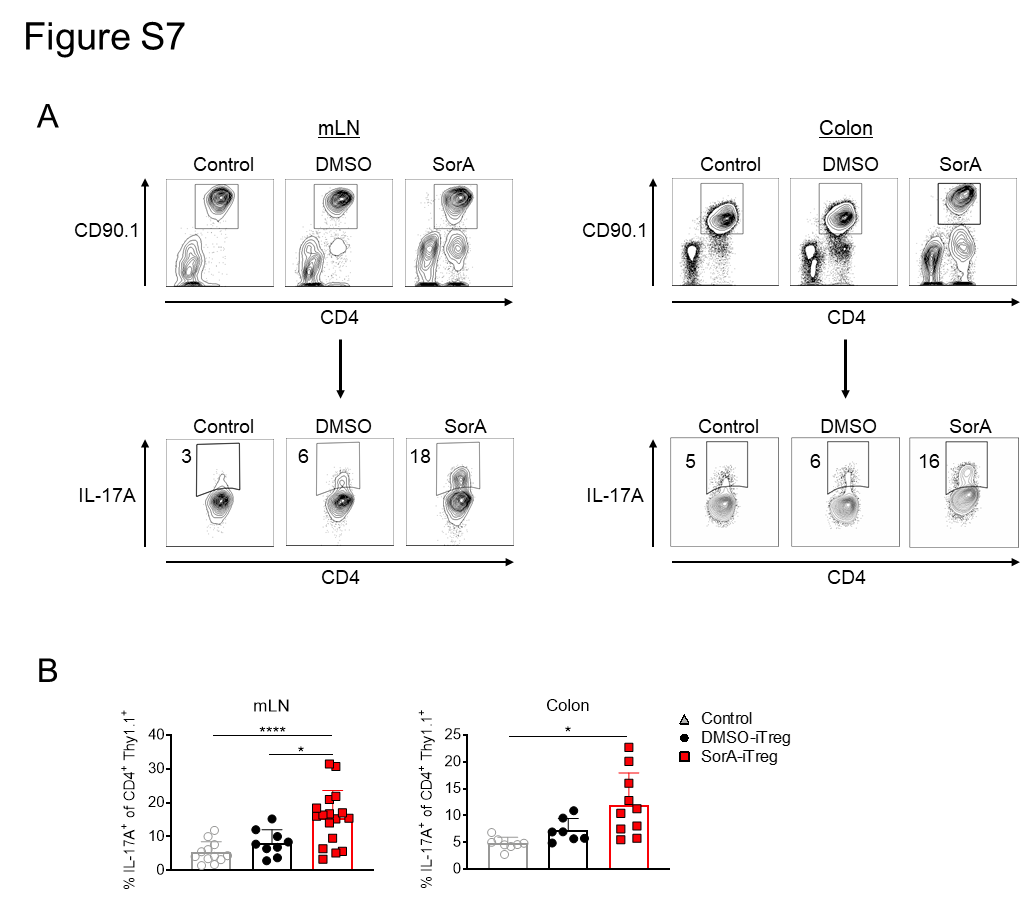
**

**Figure S7: Transfer of SorA-primed iTregs is associated with increased IL-17 production by T effector cells during colitis.** (A) Representative gating strategy for determining the cytokine production of transferred CD90.1^+^ T effector cells by flow cytometry. (B) Graphs show the frequency of IL-17-producing CD4^+^CD90.1^+^ cells in mLN and colon determined by flow cytometry. Results are pooled from two (colon) or three (mLN) independent experiments with n = 3-7 mice per group. Each symbol represents an individual mouse. Error bars show the s.d. (B). *P < 0.05, ****P < 0.0001. n.s., non-significant. One-way ANOVA with Bonferroni correction (B).
